# Earthquakes track subduction fluids from slab source to mantle wedge sink

**DOI:** 10.1126/sciadv.aav7369

**Published:** 2019-04-03

**Authors:** Felix Halpaap, Stéphane Rondenay, Alexander Perrin, Saskia Goes, Lars Ottemöller, Håkon Austrheim, Robert Shaw, Thomas Eeken

**Affiliations:** 1 Department of Earth Science, University of Bergen, Bergen, Norway.; 2 Department of Earth Science and Engineering, Imperial College London, London, UK.; 3 Physics of Geological Processes (PGP), The Njord Centre, Department of Geosciences, University of Oslo, Oslo, Norway.

## Abstract

Subducting plates release fluids as they plunge into Earth’s mantle and occasionally rupture to produce intraslab earthquakes. It is debated whether fluids and earthquakes are directly related. By combining seismic observations and geodynamic models from western Greece, and comparing across other subduction zones, we find that earthquakes effectively track the flow of fluids from their slab source at >80 km depth to their sink at shallow (<40 km) depth. Between source and sink, the fluids flow updip under a sealed plate interface, facilitating intraslab earthquakes. In some locations, the seal breaks and fluids escape through vents into the mantle wedge, thereby reducing the fluid supply and seismicity updip in the slab. The vents themselves may represent nucleation sites for larger damaging earthquakes.

## Teaser

Fluids trigger earthquakes as they flow upward along the subducted slab and escape into the overlying rocks.

## INTRODUCTION

Subduction zones, where cool lithospheric plates plunge into Earth’s hot mantle, are host to frequent earthquakes. While the largest earthquakes occur at the interface between the subducting and overriding plates, earthquakes inside the sinking plate (slab) are also abundant. How these intraslab earthquakes are produced remains poorly understood, but clues point to fluids playing an important role. Upon sinking and heating, the rocks of the subducting plate undergo phase changes whereby hydrated minerals break down into denser anhydrous phases, releasing water into the system (*1*, *2*). A leading hypothesis, referred to as dehydration embrittlement, postulates that this water triggers earthquakes at intermediate depths (50 to 300 km) by increasing pore pressure and reducing normal stress across potential planes of weakness, in a part of the system where high pressure (*P*) and temperature (*T*) conditions would normally prohibit earthquake rupture (*3*).

Despite its appeal, dehydration embrittlement poses a conundrum in cold subduction zones, where the onset of dehydration lies at depths greater than 80 km (*4*): Why is there a continuous band of earthquakes along the top of the forearc slab, i.e., the segment of the slab extending from the trench down to the onset of dehydration (*5*)? The answer may lie in a rivaling hypothesis, self-localizing thermal runaway (*6*), which suggests that rupture occurs along millimeter-thick shear zones in which the strain rate is rapidly amplified by progressive heating and weakening of the sheared rocks. This mechanism relies much less on the presence of water, but it appears to be restricted to narrow *P*-*T* conditions that do not encompass the entire forearc slab (*6*, *7*). Therefore, fluid-aided embrittlement or weakening (which need not be collocated with dehydration reactions) remains an attractive option for cold subduction zones provided that fluids can migrate updip through the subducting crust from where they are released, triggering earthquakes along their path.

Fluid flow in subduction zones has been constrained via geophysical observations and numerical models. At slab depths greater than ~80 km, most fluids appear to migrate vertically upward into the hot mantle wedge, leading to the generation of partial melts that feed the volcanic arc (*8*, *9*). At slab depths less than ~80 km, fluid distribution varies as a function of the thermal state of the system. In warm subduction zones, where the subducting crust starts to dehydrate at shallow depths, large amounts of fluid enter the cold corner of the mantle wedge (*4*). Here, temperatures are too low for melting, and fluids transform dry peridotite to a hydrated mineral assemblage containing serpentine (*5*). In cold subduction zones, the corner of the mantle wedge is usually not serpentinized, pointing to limited fluid circulation in that part of the system (*4*). Yet, episodic seismicity at the plate interface (*10*) and very low seismic velocities (*11*) suggest that at least some fluids must be present in the forearc slab. A question remains as to where these fluids come from if the only sources are dehydration reactions that occur tens of kilometers downdip.

**Fig. 1:**
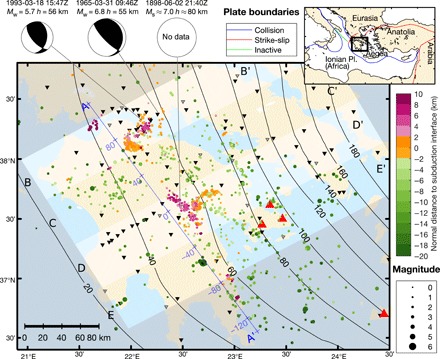
Map of the Peloponnese region of the Hellenic subduction zone. The displayed area is indicated in the plate boundary map in the inset. The map shows the seismicity from 2006 to 2017 investigated in this study (colored circles), arc volcanoes (red triangles), and the depth contours of the top of the Ionian slab [black lines: inferred interface from (*16*) with small adjustments described in the Supplementary Materials], which subducts beneath the region from southwest to northeast. Seismograph stations (inverted triangles) with more than 100 observations are displayed (filled black: waveforms used in analysis; gray outline: only picks used). Beach balls depict the focal mechanisms of three major historic earthquakes that are believed to have occurred on the plate interface at depths that are unusually large for interplate earthquakes. Vertical profiles of seismicity across sections A-A′ to E-E′ are shown in Fig. 2.

Here, we use newly relocated seismicity, in conjunction with seismic images and geodynamic models, to show that earthquakes effectively track the updip flow of fluids along the forearc slabs of cold subduction zones. Our main study area is western Greece (Fig. 1), a region where the 230–million year (Ma)–old oceanic part (*12*) of the African plate subducts beneath the Aegean microplate at a rate of 35 mm/year (*13*). Below the Peloponnese, the subduction zone is characterized by weak interface coupling (*14*), reflected historically in shallow megathrust earthquakes that do not exceed magnitude 7 (*15*), and by intermediate-depth earthquakes reaching down to a maximum depth ranging between 90 and 190 km depending on position along strike (*16*). Although the slow convergence and maximum earthquake depth suggest a thermally moderate system, the old age of the slab and a shallow subduction angle of 21° position it as a cold endmember in terms of forearc interface temperature (*8*). Western Greece is ideally suited for a seismic investigation of this scope owing to its wide onshore forearc region, which has been well instrumented to produce the comprehensive data necessary for wide-aperture imaging and earthquake relocations.

## RESULTS

**Fig. 2:**
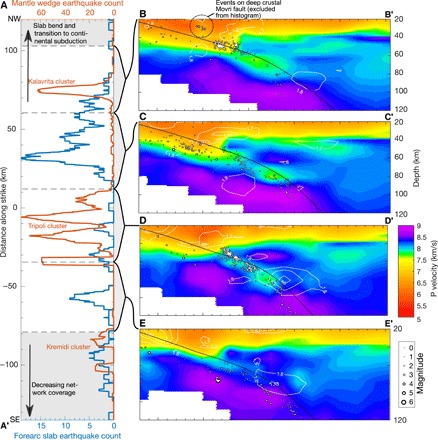
Distribution of slab earthquakes and seismic velocity structure beneath western Greece. (**A**) Number of mantle wedge earthquakes (located above the plate interface) and forearc slab earthquakes (located updip of line A-A′ in Fig. 1) along the subduction strike, calculated with a 5-km-wide moving window. The curves are anticorrelated, indicating that clusters of mantle wedge seismicity are associated with updip segments of reduced intraslab seismicity. (**B** to **E**) Body wave tomographic images from (*16*) along four cross sections (see Fig. 1 for locations) showing the low-velocity subducting crust and relocated seismicity with absolute or relative location errors of <5 km. Contour lines indicate areas with *V*_p_/*V*_s_ ratio of >1.8 and <1.7 (see fig. S2 for full *V*_p_/*V*_s_ ratio structure). High values indicate the presence of free fluid or melt, particularly in the subarc mantle, while low values point to quartz enrichment in the lower overriding crust (*18*).

We calculate earthquake locations by inverting arrival times of seismic waves recorded at seismographs across western Greece (Fig. 1) using the double-difference (DD) method with a recently published three-dimensional (3D) tomographic model of the region (*16*) for background seismic velocities. This approach yields average absolute hypocenter errors of less than 1.9 km in all directions (see Materials and Methods section). Plotted in map view (Fig. 1) and in cross section on top of the background 3D velocity model (Fig. 2), the relocated earthquakes form a single Wadati-Benioff zone (WBZ)—i.e., a dipping band of seismicity that follows the slab directly below the plate interface. However, the character of this WBZ changes along strike. Although the WBZ is fairly continuous in some profiles (Fig. 2, C and E), it exhibits local interruptions and excursions into the mantle wedge in other profiles, especially beneath the areas of Kremidi, Kalavrita, and Tripoli (Fig. 2, A, B, and D). We will focus on the Tripoli profile as it is the best sampled by our data.

**Fig. 3:**
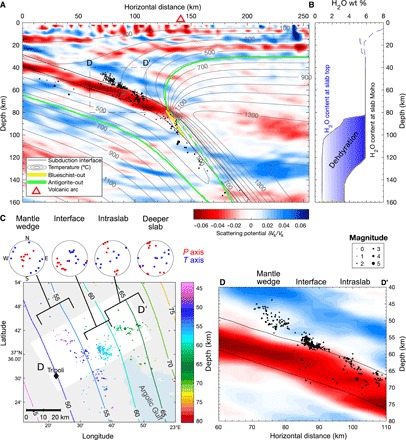
Seismic and thermal attributes of the Tripoli cluster of mantle wedge earthquakes. (**A**) Cross section (same location as Fig. 2D) of all earthquakes with location errors of <2.5 km superimposed on a teleseismic scattered wave image (*13*, *16*), with temperature contours from the geodynamic model and main dehydration reactions outlined in color (see legend). The background color scale represents scattering potential in terms of relative S-wave speed (*V*_s_) perturbations. The image resolves two main features: the hydrated subducting oceanic crust denoted by a low-velocity (red) dipping layer and the Moho of the overriding plate denoted by a downward slow-to-fast velocity (red-to-blue) contrast at a depth of ~25 km. (**B**) Water content at the top and bottom of a fully hydrated subducted crust calculated from thermal-petrologic models. No major dehydration reaction occurs before the crust reaches depths of >80 km. (**C**) Map view of the Tripoli cluster grouped by earthquake category and associated focal mechanisms (see fig. S3 for individual mechanisms). (**D**) Magnified cross section centered on the Tripoli cluster [see areas indicated D-D′ in (A) and (C)] showing earthquakes with relative location errors of <0.25 km.

To determine precisely in what part of the system the earthquakes occur beneath Tripoli, we plot the hypocenters on top of a high-resolution seismic image of the Hellenic subduction zone (Fig. 3A). The low-velocity signature of the subducting oceanic crust (−10 to −15% *V*_p_ and −8% *V*_s_ relative to the surrounding mantle) can be entirely explained by hydrated basalts and gabbros (*1*), but we cannot rule out a contribution from pore fluid being carried to depth (*17*, *18*). The implications of the latter will be further explored in Discussion. Assuming that mineral-bound water dominates the signal, the disappearance of the low-velocity subducted crust at a depth of ~90 km marks the transformation of crustal rocks into eclogites (*13*)—a process that releases up to 5 weight % (wt %) of water into the system [(*2*) and Fig. 3B]. Together with our tomographic results (Fig. 2), the high-resolution image only allows very limited (below detection level) serpentinization of the mantle wedge beneath western Greece, as expected for cold subduction systems (*4*). We identify three subclusters of enhanced earthquake activity across this image, from bottom to top (Fig. 3, C and D): a plane of intraslab earthquakes dipping at ~45° relative to the interface in which along-trench compression dominates [consistent with the prevalent mechanisms of intermediate depth earthquakes in the region (*19*)]; a dense, 5 km × 8 km (dip × strike) patch of earthquakes that appears to sit on the interface, in which interface-parallel thrusting dominates; and a more diffuse sheet of earthquakes (see fig. S1 for along-strike structure), which rises obliquely into the mantle wedge from the interface, in which trench-parallel extension dominates. In the subducting crust updip from this cluster, seismicity is virtually absent except for a few earthquakes near the subducting Moho.

**Fig. 4:**
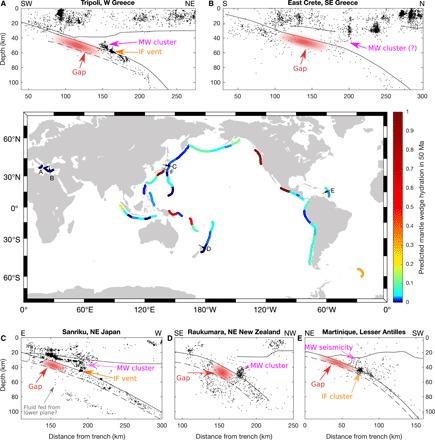
Clusters of mantle wedge seismicity. The map indicates the worldwide distribution of subduction zones and their calculated hydration state after 50 Ma of slab dehydration [data from (*1*) and (*2*)]. Observations of mantle wedge seismicity are shown in cross sections A to F. (**A**) Seismicity for the Tripoli cluster studied in this paper. (**B**) Seismicity for east Crete. (**C**) Seismicity for offshore Sanriku. (**D**) Seismicity for Raukumara. (**E**) Seismicity for Martinique in the Lesser Antilles. The exact location of cross sections and the data sources are listed in table S3 (description in Supplementary Text). A similar observation not shown here has been made onshore Chiba [east Japan, indicated as F on the map (*10*)]. The updip gap in seismicity is shaded in red. Where distinguishable, the vent marked by a cluster of seismicity on the interface (IF) is indicated, along with the cluster of mantle wedge (MW) seismicity.

Mantle wedge seismicity such as that observed beneath Tripoli is rather extraordinary but not without precedent. A handful of similar observations have been made in Japan (*10*, *20*), New Zealand (*21*), the Lesser Antilles (*22*), and Crete (*23*). Various processes have been proposed to explain the phenomenon, including (i) exotic material (e.g., piled seamounts and plume underplating) of distinct composition and high viscosity in the mantle wedge (*20*, *22*), (ii) serpentine dehydration embrittlement (*21*), and (iii) pulses of fluids released from the plate interface (*10*, *22*). Although no consensus has been reached, the similarities between these different clusters in terms of both geometry (localized clouds extending 10 to 40 km above the slab) and earthquake mechanisms (extensional) point to a common origin. There is an additional shared attribute that has received little attention until now but will be key in the following discussion: In all these regions, the subducting crust directly updip from the mantle wedge earthquakes exhibits a region of diminished seismic activity (Figs. 2A and 4).

## DISCUSSION

**Fig. 5:**
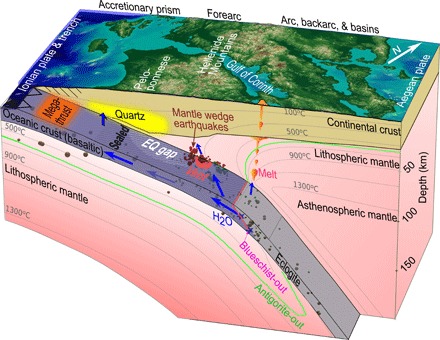
Schematic showing fluid migration paths between the sources and sinks of water in the Hellenic subduction zone. The fluids originate from dehydration reactions in the slab and flow either toward the melting zone in the mantle or updip below a sealed plate interface. Fluids that flow updip trigger earthquakes (EQ; black circles) as they migrate along the slab toward the overriding crust (where they precipitate into quartz) or if they escape via localized vents into the cold mantle wedge corner (where they cause unusual mantle wedge earthquakes). Reduced fluid flow in the subducting crust updip of these vents leads to reduced seismicity. Other subduction zones with cold interfaces and dry mantle wedges (see Fig. 4) exhibit similar fluid migration patterns.

Our seismic results, in conjunction with thermal-petrologic models constructed specifically for western Greece (see Materials and Methods), can help discriminate between the possible causes of mantle wedge earthquakes. First, we can test the serpentine dehydration hypothesis by contrasting the *P*-*T* outline of the serpentine-out reaction with the seismicity distribution (Fig. 3). This comparison shows that earthquakes occur in a portion of the system that is much too cold for deserpentinization to occur. Thus, the deserpentinization hypothesis is not supported by our results. Second, we can also reject the hypothesis of large bodies of exotic material in the mantle wedge, as these would cause velocity perturbations that are not supported by the seismic images (Figs. 2 and 3), which show a fairly uniform, high-velocity mantle wedge beneath western Greece. Our results point instead to mantle wedge seismicity as being due to fluids released from the subducted slab (*10*). They also tell a broader story of fluid transit from source to sink in cold subduction zones (Fig. 5), which we shall now explore further with the aid of our thermal-petrologic models.

The deepest slab earthquakes appear to mark the primary locus of mineral-sourced fluids, associated with the blueschist-eclogite transition in the crust and the antigorite-out reaction in the mantle (*5*, *24*). Our thermal-petrologic models (Fig. 3B) show that, for western Greece, these reactions occur at depths of 80 to 140 km and 80 to 200 km, respectively, with limited sources of fluids present at shallower depths. These reactions occur along isotherms that are oblique to the dip of the slab, matching well the obliquity observed between seismicity and slab structure (Fig. 3A). Major fluid production and release at this depth is also supported by the tomographically imaged high *V*_p_/*V*_s_ ratio in the overlying subarc mantle wedge [Fig. 2D, fig. S3, and (*16*)].

While most fluids escape directly upward into the mantle wedge through a plate interface damaged by metamorphic reactions (*25*), some must make their way updip through the slab. This updip flow can occur under an intact plate interface that remains sealed owing to shear-induced grain size reduction (*9*, *18*, *25*) and/or along a direction of minimum effective pressure that follows the slab (*26*). The latter has been invoked to explain intraslab seismicity (*11*, *26*) and could produce the zones of weakness required to facilitate rupture in the Hellenic forearc slab updip of 80 km depth. However, simply observing intraslab seismicity above 80 km is insufficient to favor this fluid-aided weakening over alternative rupture processes. What establishes fluid as an essential player is the fact that, at some locations, the seismicity deflects from the slab into the mantle wedge, leaving regions of considerably reduced seismicity updip of the point of deflection. This points to a scenario in which the earthquake-inducing fluids, during their updip migration, encounter a vent at the interface that diverts them into the mantle wedge. The slab segment directly updip of the vent experiences diminished fluid circulation and is thus less prone to rupture. Diminished fluid flow may also be responsible for the weakened seismic signature of the subducted crust in the aseismic segment (see Fig. 3A; between 20- and 60-km horizontal distance), although this perturbation is small enough that it could be an artefact due to variations in ray coverage. In addition, this mechanism allows the potential presence of pore fluids carried down with the slab (*18*), as mentioned in Results. In the aseismic segments of the slab, the pore fluid pressure would presumably be at steady state but would be lacking the transient perturbations caused by migrating fluids that are necessary to trigger seismic rupture (*17*). Further updip, the intraslab seismicity picks up again in all subduction zones surveyed (Fig. 4 and Supplementary Text) at depths of <30 to 50 km corresponding to the shallow earthquake range, where rheological conditions allow normal brittle failure.

The process by which interface vents can channel fluids from the slab into the mantle wedge was the subject of a recent seismological study in Japan (*10*). In the area of that seismicity cluster, evidence points to fluids that accumulate under a low-permeability seal at the interface and periodically break through the seal in response to slow-slip events that occur at the base of the locked zone [also seen in Cascadia (*18*)]. The fluids escape into the mantle wedge, where they cause transient clouds of earthquakes by temporarily increasing pore-fluid pressures and thus promoting brittle behavior. A similar process could apply to Greece, although differences in subduction dynamics compared to eastern Japan appear to influence the flux of fluids. The mantle wedge earthquakes below Tripoli lack the annual cyclicity of their Japanese counterparts (fig. S2). This difference likely stems from the contrast in convergence rate between Japan [82.7 mm/year (*8*)] and Greece [35 mm/year (*13*)], which could lead to much longer (factor of ~6; see Supplementary Text) cycles in Greece.

At regional scales, the spatial distribution of the seismicity clusters provides additional clues about the origin of the interface vents. Below western Greece and other similar subduction segments, the clusters of interface seismicity occur systematically within a narrow depth range of 45 to 60 km {Greece, 45 to 60 km [(*23*) and this paper], northeast Japan, ~50 km (*20*); east Japan, 42 to 60 km (10); New Zealand, ~55 km (*21*)} and are usually separated by 30 to 100 km along strike (*20*, *21*). This suggests that the location of the fluid vents is not controlled by structural features of the slab making their way down the interface, as this would imply that clusters can occur over a much wider range of depths and with random along-strike spacing. Instead, the exit point of fluids must be controlled by large-scale dynamical, mechanical, and geometrical properties of the system. For example, fluid focusing could arise from anisotropic permeability in the subducted crust and at the interface, whereby fluids flow preferentially toward a focal point (*26*), and/or from 3D undulation of the slab, whereby fluids are channeled by along-strike bends in the slab (*27*). There is evidence that these two mechanisms are operating in Greece: (i) Anisotropic permeability is supported by apparent dipping planes of seismicity in the subducted crust, which point to fluids preferentially migrating along fault planes oblique to the interface; (ii) seismic images indicate that the slab is deformed beneath most of the Peloponnese (*15*, *16*), providing along-strike 3D undulations that may channel the fluids.

The overall frictional behavior across the patches of interface seismicity is unknown. Consider a scenario where the patches represent locked asperities (with localized points of slip) surrounded by a generally stable sliding interface (*20*). If weakened enough by accumulated fluids, the whole patch could sometimes rupture, producing large, damaging earthquakes. For the Tripoli cluster, we calculate that the interface seismicity has generated a cumulative seismic moment of $6.8_{-0.3}^{+9.4}$ × 10^21^ dyne∙cm over 11 years (see Materials and Methods). Given that the observed patch area of 5 × 8 km^2^ represents the minimum slip surface, this would imply that less than ~0.4% of the patch has ruptured over the period. If the asperity is locked, we could then expect an earthquake of *M*_w_ (moment magnitude) = 5.9, on average, every 40 years. This is of comparable magnitude to the *M*_w_ = 5.7 subduction thrust earthquake that occurred at a depth of 56 km on 18 March 1993 (*14*), less than 10 km from the Kalavrita cluster (Fig. 1). A considerably larger *M*_w_ = 6.8 subduction thrust event occurred near the same location at a depth of 55 km on 31 March 1965 (*14*, *28*). Its radiation characteristics and intensity (VII on MSK-64 scale) match that of the *M*_s_ (surface wave magnitude) ~ 7 historical earthquake of 2 June 1898, inferred to have occurred at intermediate depth below the Tripoli region (*28*, *29*). Both of these large events would have ruptured an area on the interface that is an order of magnitude greater than the currently active patch. Still, these earthquakes may have nucleated in the active patch and ruptured far into velocity-strengthening regions of the interface (*30*). Mapping of these patches of interface seismicity could therefore help identify regions of enhanced seismic risk.

Our analysis suggests that, for cold subduction zones, the bulk of intermediate depth earthquakes in the 40- to 80-km depth range is due to fluids that migrate updip along the slab. However, the question about the ultimate fate of these fluids remains. With no evidence that detectable volumes reach the surface, the fluids must react with, and become absorbed in, the overriding crust and mantle wedge. For the overriding crust, this is supported by observations in several subduction zones, including Greece, of low *V*_p_/*V*_s_ ratio regions, which have been attributed to quartz precipitation from slab-derived fluids (*18*). For the mantle wedge, water can easily become absorbed via serpentinization of cold peridotitic rocks. However, seismic evidence suggests only a thin layer of serpentine above the interface in western Greece (*4*, *13*, *16*, *31*). This is also consistent with the occurrence of earthquakes in the wedge, as too much serpentine (>10%) would weaken the rocks to levels that preclude brittle failure (*32*). This limited serpentinization is not inconsistent with the proposed injection of water into the mantle wedge via interface vents. To achieve levels of serpentinization that prevent brittle failure anywhere in the mantle wedge, a minimum of 40 Ma of steady venting is needed (see Materials and Methods). This sustained venting is unrealistic in subduction zones—as these are often not at steady state (especially western Greece), the interface vents are likely sporadic features. Better constraints on the transient nature of interface vents will clarify how the flow of earthquake-inducing fluids evolves, thus improving our ability to assess seismic hazards in subduction systems.

## MATERIALS AND METHODS

### Data sources and overview of the new earthquake catalog

We collected earthquake and waveform data from various sources to create a catalog of deep earthquakes (depth greater than 35 km) below central Greece. This new catalog has higher resolution and provides a complete inventory to a lower magnitude threshold (~1.9) than previous catalogs published for the region.

The basis for our catalog is a set of 914 deep events that were originally reported by the International Seismological Centre (ISC) and the National Observatory of Athens (NOA) and were used in a recent tomographic study (*16*). To complement this original dataset, we reanalyzed publicly available waveform data downloaded through the International Federation of Digital Seismograph Networks (FDSN) web services. This allows us to detect additional events and obtain new phase arrival picks over the 2006–2017 period for stations listed in table S1.

With our reanalysis, we supplemented the original dataset with 1288 new events, for a total of 2172 deep earthquakes beneath the region. The contribution of each network to this new catalog of events is as follows: 317 additional events published by NOA for 2016–2017 (*33*), which were reevaluated; 689 events recorded by the combined Medusa-Egelados networks between June 2006 and October 2007, concentrated in the region of the Tripoli cluster; and 282 more events from the Tripoli cluster that we detected on permanent stations in Greece over the period 2008–2017. All the events analyzed here have picks from at least six stations (mean: 25 stations), yielding a dataset of 52,713 P-arrival picks and 37,310 S-arrival picks (previously: 29,399 P and 13,179 S) from 379 stations. Of these stations, 289 had 10 or more observations (147 stations with *n* ≥ 100 and 27 stations with *n* ≥ 1000). In the following sections, we describe the analyses that were performed to detect and relocate the deep earthquakes.

### Earthquake detection

The catalogs of deep earthquakes that were available for western Greece before this study were complete only down to magnitudes of ~2.8 (see fig. S5A), and very few deep events of magnitude smaller than 3 were detected in the seismicity clusters of Kalavrita, Tripoli, and Kremidi. Our goal here is to expand these catalogs to a lower completeness magnitude threshold and to increase their resolution by exploiting the comprehensive station coverage afforded by combining the networks listed in table S1. We take into account the fact that station coverage was particularly dense in the Peloponnese between 2006 and 2007, owing to the operation of the Medusa and Egelados networks during that time. With this in mind, we developed a three-step earthquake detection workflow based on waveform matching to build our newly improved catalog.

First, we built a suite of robust earthquake waveform templates to help detect small magnitude events below western Greece, focusing initially on the 2006–2007 period of higher station coverage. A robust template must stem from an earthquake that has a verified depth of >35 km (see section below), with recordings from at least 12 channels from a group of stations—i.e., P and S waveforms recorded at a single station count for two channels. Selected templates must have a signal-to-noise ratio of >1.2 in a chosen frequency band, which varies as the search is expanded. We started by looking for earthquakes that meet these criteria in the catalog of (*16*) and found a set of 30 events that could be used as templates for the 2006–2007 period. Thirty templates is a somewhat restrictive number when trying to detect earthquakes in the slab below the core of the network (~200 km × 110 km, dip × strike), so we expanded our search by using a short-term average/long-term average network detector on a selection of low-noise stations from networks XS (S005 to S023), Z3 (PE01, PE02, PE07, IDHR), and HL (LTK), which were all running during the same time window. A detection is made when the average amplitude over a 30-s window exceeds the average amplitude of a 120-s window at each station by a factor of 6 and when at least 12 of the 27 stations are triggered within a 10-s window. From 700 such detections, we identified ~50 previously undetected deep (>35 km) earthquakes that occurred within the dimensions of the network. This gave us an initial suite of 80 templates that can be used for further detection.

Second, we used our initial suite of templates to run a matched filter network detector on the data of stations S009 to S014, which best sample the Tripoli cluster and afford the highest signal-to-noise ratio. The template matching was done with the EQcorrscan 0.2.7 software package (*34*) on the template envelopes filtered between 1.5 and 6.0 Hz. We used the envelopes because they describe any earthquake from a similar source region, as reflected by a distinct separation between P and S phases, rather than events with similar mechanism, which would restrict our search to repeating earthquakes. With this approach, we detected another 259 deep events, which were all manually picked and verified for quality control.

Last, we expanded our search to earthquakes that occurred over the entire available time span of 2006–2017. To do so, we ran the EQcorrscan template matching software for all stations that were operating during that time in the northeastern Peloponnese and Attica region. We used a suite of templates that comprised the templates and detected events from step 2 (339) plus 255 events from the NOA catalog, for a total of 594 templates. Here, the template matching was done on the waveforms instead of the envelopes. The waveforms were 7 s long (starting 0.2 s before the P or S pick) and filtered between 2.5 and 8.0 Hz. A detection triggers when the median average deviation of the cross-correlation (CC) sum across channels is >8, upon which the program picks an arrival on those channels where the highest single-channel CC exceeds 0.4 in a 0.8-s window around the detection. We retained events with at least six P picks for manual review. Only events located within −60/+50 km of the profile in Fig. 3A were retained. This returns an additional 646 events for the 2006–2017 period.

Through the procedure described above, we added 941 events to the catalog of (*16*), which, together with the 317 newly verified deep events of the NOA catalog 2016–2017, result in a catalog with a total of 2172 events. For the period of 2006–2017, this catalog is complete to a magnitude threshold of 1.9 (fig. S5B), owing mainly to the quality control that allowed us to identify and reject events from the NOA catalog that were located in the upper plate. Within the vicinity of the Tripoli cluster, our detection procedure yields a data subset (plotted in Fig. 3A) that is complete to a lower magnitude threshold of 1.1, as shown in fig. S5C.

### Earthquake relocation

To determine precise earthquake hypocenters, we adopted a relocation procedure that comprises three stages: (i) inversion in the 1D background velocity model obtained in (*16*), (ii) inversion in the 3D background velocity model obtained in (*16*), and (iii) relocation with the DD method (*35*). Stages (i) and (ii) closely followed the methodology of (*16*), except that, here, we fixed the velocity model to those obtained in that study—i.e., the velocity models were not updated during the relocation. Below, we discuss the outcome of each stage in more detail.

In stage (i), we computed earthquake locations by least-squares inversion of P- and S-arrival times in a 1D model. We performed picking in Seisan (*36*), and the relocation was done with the program Hypocenter (*37*) included in Seisan. For each event, we visually checked that picks were correctly assigned and removed picks with residuals larger than 6 s. We estimated absolute location errors from the solution variance (arrival time misfit) as described in (*16*, *37*). The hypocenter solutions obtained at this stage have average location errors of 5.1 km in the horizontal direction and 7.5 km in the vertical direction. The results were used to identify events with deep origin (>35 km depth, taking into account vertical error bars) that will be retained for further processing.

In stage (ii), we relocated individual earthquakes by iterative least-squares inversion of P- and S-arrival times in a 3D model to obtain more accurate locations for the events retained in the previous stage. The initial locations used for the inversion are those obtained in stage (i). We calculated arrival times in the 3D velocity model with SIMULR16 (*38*), using the same ray tracer, parametrization, and distance- and residual-dependent weighting scheme as in (*16*). This inversion yields hypocenter solutions with considerably smaller absolute location errors averaging 1.3 km in the horizontal direction and 1.8 km in the vertical direction.

In stage (iii), we relocated events by minimizing differential arrival times between event pairs, which enhances the resolution of relative hypocenters for clustered events. The differential arrival times used as inputs were obtained via both catalog hypocenters and CCs of seismic waveforms. A first list of inputs was built by compiling differential arrival times for all pairs of catalog hypocenters from stage (ii) that are less than 20 km apart. This results in 1,851,141 P-differential arrival times and 1,695,296 S-differential arrival times from 265 stations. We then identified pairs from the catalog list that are less than 12 km apart. For these, we computed a second list of differential arrival times by cross-correlating waveforms with the EQcorrscan package and the ObsPy toolbox (*39*). The waveforms we used for CC were 1.0 s long, starting 0.3 s before the P and S phases, and were band pass–filtered between 2.5 and 8 Hz. To retain a differential time pick, we required a normalized CC coefficient of at least 0.7 and a resulting value that differs by less than 0.4 s from the catalog value. This yields a second list of 173,173 P-differential arrival times and 142,128 S-differential arrival times from 133 stations. The differential arrival times from the two lists were then inverted simultaneously via a DD algorithm with the hypoDD program [version 2.1b (*33*)].

HypoDD’s DD algorithm involves an iterative inversion in which we can introduce a stricter weighting on the input data at each new iteration. This was done by progressively increasing the weight for CC differential times (from 0.02 to 1), by reducing the maximum distance across which event pairs are compared (from 12 km down to 3 km for CC-based inputs), and by removing data outliers (from a complete dataset in the first iteration to the removal of data points with residuals larger than six times the standard deviation (SD) of all residuals in the final iteration). In the final iteration, we were thus left with 51% of catalog and 32% of CC differential arrival times to estimate the relative locations of clustered hypocenters. At this stage, we allowed event pairs to form a cluster when they are connected through at least eight catalog differential times and eight CC differential times. To address the variable data coverage during the entire time period (2006–2017), we varied inversion parameters and verified that hypocenters relocate consistently for overlapping data subsets. This led us to adopt a relatively high damping value of 600 to achieve a reasonable system condition number of 40 to 60 for the entire dataset. We found that active structures outlined by hypocenters from the high-coverage period (June 2006 to October 2007 for the Tripoli cluster) were also well constrained and appeared more complete when including hypocenters from the complete time period.

The results of the DD relocation are presented in Figs. 1 to 3 and are available in external data file S2. As there are no large gaps in seismicity that would interrupt the clustering chain within the slab, most slab seismicity was contained within one large cluster. This remains true regardless of the existence of localized seismicity gaps updip of the interface vents, as hypocenters remain connected away from these zones. Separate clusters occurred, e.g., at large depth and in the deep overriding crust (below the Gulf of Patras). Shallower earthquakes in the overriding crust were not processed. As the DD relocation mainly improves relative hypocenter locations, here, we estimated relative errors [rather than absolute errors as in stages (i) and (ii)] through jackknife resampling of the dataset (*35*, *40*). For this, we reran the DD inversion 1000 times with a reduced dataset in which 10% of the differential arrival times were randomly removed. The errors were then estimated with the general “delete-j” jackknife estimator described in (*40*). We find that, on average, the relative location error is 0.19 km in the horizontal direction and 0.21 km in the vertical direction. The relative errors of earthquakes within ±50 km of the cross section in Fig. 3A are shown in fig. S7.

### Focal mechanisms

We calculated focal mechanisms from first motion polarities and selected the best solution based on the misfit of amplitude ratios between P and S arrivals. This was done using the program FocMec (*41*). The polarities were picked manually on the vertical channel. Solutions were calculated only for events that have at least 10 consistent polarity picks (the average was 23 picks). The amplitude ratios were automatically measured in the frequency domain in Seisan. We adopted a two-step workflow to compute the focal mechanisms. In the first step, we used FocMec to find an approximate solution based exclusively on polarities, searching the parameter space for strike, dip, and rake values that best fit the polarities. When we found concentrated groups of solutions (i.e., solutions where the *P* and *T* axes fall in small, ~^1^/_10_ areas of the lower hemisphere projection), we proceeded to the second step in which we constrained the solutions further by fitting observed amplitude ratios to theoretical predictions. For these fits, we imposed two restrictions to help identify robust solutions: (i) The number of amplitude ratios, where observation and prediction do not match, should not exceed 25 to 50% of the total number of measurements and (ii) the maximum amplitude ratio error, i.e., the difference between the measured and predicted amplitude ratio, should not exceed 30 to 40%. We aimed to choose these values such that they yield less than ~30 solutions when searching the parameter space in steps of 1° to 2°. In the case where an earthquake has a large number of polarity picks (≳20), this second step requires us to tolerate more polarity errors than in the first step, since we may be fitting more polarities close to the nodal planes. However, we only allow the solutions from step 2 to refine those from step 1 and do not accept solutions that differ markedly between the two steps.

Here, we solved for 81 focal mechanisms with the approach described above. This initial list was complemented with 21 solutions from the literature (see table S2). This resulted in a catalog of 85 earthquakes with one or more focal mechanism solutions. Figure S4 shows 38 of these focal mechanisms for earthquakes that occurred within section C of Fig. 1 (see also Fig. 3A).

### Thermal and phase stability modeling

We calculated temperatures for the subduction zone and flow in the mantle wedge (and below the subducting plate) as in the study of Perrin *et al*. (*42*) by solving the coupled Stokes and energy equations in Boussinesq approximation using the code Fluidity (*43*). We constrained the models by kinematically prescribing the subducting plate, with the plate interface interpreted from the scattered wave image in Fig. 3A. Beyond the depth to which the image recovers a sharp velocity contrast at the top of the subducting crust, we traced the lower-to-higher (from top to bottom) velocity contrast in the tomographic image in fig. S6A, which we then extended linearly to depth. At an approximate angle of 45°, this trend agrees well with the dip of the slab imaged in mantle-scale tomography [(*44*) and references therein]. The top 10 km of the subducting plate has a set velocity of 35 mm/year (*13*). Because of the composite temperature- and pressure-dependent dislocation-diffusion creep rheology [all parameters as in the study of Perrin *et al*. (*42*)], the slab is strong and is pulled down by the prescribed top layer, while the mantle wedge is weak and flows in response to the sinking slab. The top 50 km of the upper plate and a 5-km-thin layer above the slab down to the decoupling depth (i.e., the depth beyond which mantle wedge and subducting plate move at the same velocity) were kept fixed.

The thermal structure of the incoming plate was set to a half-space cooling structure for a 90-Ma-old plate. The initial thermal structure of the upper plate was that of a 10-Ma-old lithosphere, and models were run for 50 Ma, long enough for slab temperatures to reach a quasi-steady state, so that the final thermal thickness of the upper plate is as for a thermal age of 60 Ma. All other boundary and initial conditions are as in the study of Perrin *et al*. (*42*).

We tested models with varying decoupling depth and compared imaged seismic structures with calculated seismic structures that we determined from the thermal-petrologic models. On the basis of the comparison, we converged toward a decoupling depth of 80 km, which is the same as that preferred in global studies (*45*). The favored model (fig. S6, D to F) matches the seismic images (fig. S6, A to C) in terms of the depth extent of the low-velocity crust (see fig. S6, C and F). Moreover, the locations of the main dehydration loci and volcanic arc correspond to those where a high *V*_p_/*V*_s_ anomaly occurs in the hot part of the mantle wedge and upper plate (see fig. S6, B and E).

To construct the synthetic seismic images, we first assigned different mineralogic compositions to different parts of the model and then calculated phase stability fields in each part. We assigned a hydrous mid-ocean ridge basalt (MORB) to the top 8 km of the slab (based on the apparent thickness of the low-velocity crust in the scattered wave images), a 27-km-thick layer of harzburgite below, and a depleted MORB-source mantle (DMM) composition everywhere else except in the overriding continental crust. We then mapped phase stability fields and water contents that can be held at each pressure-temperature point in the model. We used the Holland and Powell database (*46*) for hydrous MORB, hydrous harzburgite, and hydrous peridotite, implemented in Perple_X (*47*) as database hp02. Compositions for DMM were from Hacker and Abers (*48*) and those for harzburgite and MORB were from Xu *et al*. (*49*). We used the same set of solid solution models as that of Hacker and Abers (*48*) to compute phase stability fields. Figure 3B displays the water content of a saturated MORB along the geotherms at the slab top and slab Moho computed in this way. Dehydration depths do not change substantially with different water contents.

Likewise, we computed phase compositions and calculated seismic velocities at each pressure-temperature point, following the same procedure of Eeken *et al*. (*50*). Parameters for the equation of state were from Abers and Hacker (*51*), with the addition of a temperature-pressure– and free water–dependent experimental attenuation from model Qg, as in the study of Goes *et al*. (*52*). An exception was made for the upper 30 km of the overriding plate, where we assumed a seismic velocity corresponding to continental crust [platform profile from CRUST2.0 (*53*)]. Figure S6 (D to F) shows the seismic structures calculated as described above for a hydrated mantle wedge (3000 H/10^6^ Si), a subducting crust containing 4 wt % water, a dry harzburgite layer, and a dry DMM in the rest of the slab mantle, as well as in the mantle below the slab.

### Estimation of coseismic slip on the interface patch

Below Tripoli, subduction thrust earthquakes outline a patch of seismic activity at depths of 54 to 60 km on the plate interface (Fig. 3, C and D)—a depth at which the interface is usually assumed to slip aseismically. The density of earthquakes suggests that the whole patch acts as one large asperity, which would imply that the whole 8 km × 5 km = 40 km^2^ area outlined by the earthquakes can slip coseismically. However, an alternative model is that most of the patch slips aseismically, with only very small parts slipping coseismically, e.g., along narrow fluid pathways. To better assess the implications of these models, we estimated the area of the patch that slipped coseismically during the 11 years of seismic observations in western Greece. We then compared our estimated value to the total size of the patch and investigated whether it is consistent with the magnitude of large historical earthquakes.

To carry out our estimation, we selected all earthquakes from the time period of June 2006 to December 2017 that are clustered in the patch. Those are earthquakes that satisfy the following location attributes, in reference to the cross section in Fig. 3: (i) between 83.5 and 93.5 km along the horizontal distance axis, (ii) less than 25 km along-strike offset from the cross section, and (iii) between 54 and 60 km depth. We assumed that all these earthquakes occurred on the interface. This assumption is reasonable despite the fact that we do not have focal mechanism estimates for every earthquake, as the patch is distinguishable from clustered seismicity updip/downdip in the slab and above in the mantle wedge. Moreover, the high-resolution relocation shows that these earthquakes align on a well-confined plane that parallels the interface imaged by teleseismic scattered waves. All 399 earthquakes selected through this process are well located, with an average relative hypocentral error of 0.1 km horizontally and 0.17 km vertically, with only five earthquakes exhibiting a vertical error greater than 1 km and at most 2.3 km (fig. S7). Of these 399 earthquakes, 140 have magnitudes of ≥1.1, the magnitude down to which the earthquake catalog for the Tripoli cluster appears complete (see fig. S5C).

For each of these earthquakes, we estimated the interface fault area ruptured (*A*, in cm^2^) based on its relationship to the seismic moment (*M*_0_, in dyne∙cm), following the empirical relationship of Nadeau and Johnson (*54*)

$$\text{log}(A)=-9.12\pm 0.16+(0.83\pm 0.009)*\text{log}(M_{0})$$ (1)

This relationship was originally derived from shallow repeating earthquake data in California, but it has also been used for the same type of subduction plate interface earthquake as those investigated here (*55*). We estimated the seismic moment from the following *M*_w_ relationship (*56*)

$$\text{log}\;M_{0}=M_{w}+16.1$$ (2)

Here, we used local magnitudes, *M*_L_, which we determined in a consistent manner for all earthquakes after June 2006 according to a local Greek magnitude scale (*57*), calibrated to be equivalent to the moment magnitude. Rearranged for implementation in Seisan, this magnitude is defined as follows

$$M_{L}=\;\text{log}\;U+(1.319\pm 0.024)\cdot \text{log}\;R+(0.0226\pm 0.0017)\cdot R-2.546+c_{i}$$ (3)

where *U* (in nm) is the average of the maximum zero-peak amplitudes on the two horizontal channels, *R* (in km) is the hypocentral distance, and *c*_*i*_ is a station correction factor determined for all permanent stations of the Hellenic Unified Seismic Network [value between −0.48 and +0.38 (*57*)].

On the basis of Eqs. 1 to 3, we found that the sum of fault area that slipped in the 399 earthquakes is $A=0.15_{-0.08}^{+0.47}$ km^2^, which is less than 0.4% of the 40-km^2^ patch area outlined by the interface earthquakes. When we excluded earthquakes below the completeness magnitude, the estimate of the coseismic active slip area decreased by less than 2%. This indicates that our estimate relies predominantly on the 140 earthquakes with magnitudes of ≥1.1, and therefore, the risk of underestimation is very low. The area of coseismic slip may be even smaller than what we estimated, as we do not account for the fact that some earthquakes may have ruptured the same part of the patch multiple times. Regardless, our results suggest that only a minor area of the patch slipped between 2006 and 2017. For slip to occur on the whole patch of 40 km^2^ at once, Eq. 1 requires an earthquake with a seismic moment *M*_0_ = 9.25 × 10^24^ dyne∙cm, equivalent to *M*_L_ = 5.9.

We may now ask ourselves how often the entire patch would rupture, provided that all slip in the patch is coseismic. Conveniently for us, Nadeau and Johnson (*54*) did this estimation on the Parkfield segment of the San Andreas fault, where the relative plate motion of 33 mm/year is similar to that of the Western Hellenic subduction zone (35 mm/year). We can therefore estimate the recurrence rate of a *M*_L_= 5.9 earthquake by applying the relationship

$$t=10^{4.85+0.17*\text{log}M_{0}}$$ (4)

where *t* is the earthquake recurrence time. For a seismic moment of *M*_0_= 9.25 × 10^24^ dyne∙cm, we estimate that a *M*_*L*_= 5.9 earthquake would have a recurrence interval of 39.4 ≈ 40 years. Unfortunately, it is not simple to test this recurrence interval against available earthquake catalogs. Although a catalog with adequate completeness magnitude exists for the last ~110 years in Greece (*58*), the challenge lies in identifying other earthquakes that occurred on the subduction thrust patch rather than in the slab. Tackling this challenge would require a reduction of hypocentral errors in these long-term catalogs and moment tensor solutions for all candidate events. Nevertheless, as mentioned in the main text, the estimated magnitude associated with a full-patch rupture matches that of at least three intermediate depth earthquakes that have occurred over the past ~120 years in regions where we have detected clusters of interface earthquakes. This points to a high likelihood that the interface patches sometimes slip coseismically along their entire surface area.

### Mantle wedge hydration rate

Our model of fluid flow in subduction zones suggests that hydrous fluids are locally vented from the slab into the cold mantle wedge corner. However, we know that, when water enters the cold mantle wedge corner, it hydrates peridotites to form serpentine (*4*). This reaction causes an abrupt decrease in the strength of mantle rocks when the degree of serpentinization exceeds ~10% (*32*). This weakening inhibits seismic failure and makes place for ductile deformation, even at low temperatures. The presence of earthquakes in the mantle wedge thus tells us that there can only be limited serpentinization, i.e., less than 10%, beneath western Greece. To verify that these levels of serpentinization are realistic, we estimated the mantle wedge hydration rate for our study area.

First, we considered the amount of hydrous fluids vented into the corner of the mantle wedge. Comparing the along-strike width of the interface patch (8 km) with that of the associated seismicity gap (~50 km), we can assume that the fluid flow is focused by a factor of 6 along strike. This implies that all fluids produced from dehydration reactions within the slab over a 50-km-wide section are channeled through an 8-km-wide section of interface and into the overlying mantle wedge. We consider a fully hydrated (7.8 wt % H_2_O; Fig. 3B), 8-km-thick basaltic crust with a density of 3100 kg/m^3^ (*1*) subducting at 35 mm/year, which loses 5 wt % H_2_O (compared to its total mass) during the main dehydration pulse at depths of 80 to 140 km. This results in a water production rate of 6.8 × 10^5^ kg m^−1^ year^−1^. Simply channeling all this water updip and through the slab vent would result in a water discharge of 2.2 × 10^9^ kg/year, which means that the water flux through the slab vent (40 km^2^) could be as large as 90 kg m^−2^ year^−1^.

Second, we estimated a lower and upper bound for the time required to hydrate the mantle wedge by considering two cases: (i) hydrating only the volume of mantle wedge that is currently active seismically, with the highest possible water production rate as estimated above, and (ii) hydrating the complete section of mantle wedge in which serpentine is stable (i.e., the cold corner) with a lower, more realistic water production rate. Starting with the lower bound estimate, we note that the seismically active volume of the mantle wedge corner is relatively small. It extends 12 km along dip, by 50 km along strike, and by up to 10 km vertically, with seismicity ceasing ~10 km below the overriding Moho. This creates a complete volume of 12 km × 50 km × 10 km = 6 × 10^12^ m^3^ with a cross section of 120 km^2^. According to the calculations of Abers *et al*. (*4*), this translates into an H_2_O capacity of 2.0 × 10^15^ kg within the active volume. Purely hydrating this seismically active volume to 10% of the capacity would thus take at least ~0.1 Ma. However, this result is based on an upper limit of dehydration and hydration rate. In reality, the volume of water carried down by the slab is likely an order of magnitude lower than what we estimated above, owing to the layered structure and incomplete hydration of the incoming crust (*8*). Moreover, only a small fraction of dehydration fluids may channel updip, while a larger fraction migrates toward the arc. Consequently, when we consider a realistic level of initial slab hydration as in the study of van Keken *et al*. (*8*), i.e., 10 times lower than above, and only 50% of fluid channeled updip, the water discharge rate through the vent is 1.1 × 10^8^ kg/year. In this scenario, it would now take ~2 Ma to hydrate the seismically active volume to 10%, but to calculate the upper bound for hydration time, we must consider hydration in the entire extent of the mantle wedge where serpentine is stable. This region measures 2726 km^2^ in cross section, translating to a volume of 2726 × 50 km^3^ = 1.36 × 10^14^ m^3^ when considering the same 50-km along-strike section as above. With our more conservative estimates of water discharge (1.1 × 10^8^ kg/year), it would thus take 42.7 Ma to serpentinize the entire cold portion of the mantle wedge by 10%.

## Supplementary Material

http://advances.sciencemag.org/cgi/content/full/5/4/eaav7369/DC1

Download PDF

## SUPPLEMENTARY MATERIALS

Supplementary material for this article is available at http://advances.sciencemag.org/cgi/content/full/5/4/eaav7369/DC1 Supplementary Text Mantle wedge seismicity in other subduction zones Episodicity of mantle wedge earthquakes and velocity of fluid migration Fig. S1. Along-trench profiles of mantle wedge seismicity and P-velocity structure, plotted as seen from the trench. Fig. S2. Temporal evolution of seismicity in the Tripoli cluster. Fig. S3. P-velocity to S-velocity (*V*_p_/*V*_s_) ratio structure beneath western Greece. Fig. S4. Focal mechanisms of the Tripoli cluster. Fig. S5. Estimates of completeness magnitude, *M*_c_, for various catalogs of deep earthquakes (>35 km) below western Greece. Fig. S6. Comparison of seismic images and calculated seismic structure along the cross section of Fig. 1D. Fig. S7. Hypocenters displayed with their relative location errors. Fig. S8. Earthquake distribution and electric resistivity structure below the Peloponnese. Table S1. Seismograph networks from western Greece used in the waveform processing. Table S2. Focal mechanism solutions of deep earthquakes in the Western Hellenic subduction zone. Table S3. Locations of mantle wedge seismicity displayed in cross sections in Fig. 4. External Data file S1. Deep earthquake hypocenters in Greece. External Data file S2. Deep earthquake focal mechanisms in Greece. External Data file S3. Earthquake arrival time picks. External Data file S4. Model of the subduction plate interface. External Data file S5. Thermal structure model of the subduction zone. References (*59*–*81*)

## References

[1] Hacker B. R., Abers G. A., Peacock S. M., Subduction factory 1. Theoretical mineralogy, densities, seismic wave speeds, and H_2_O contents. J. Geophys. Res. 108, 2029 (2003).

[2] Schmidt M. W., Poli S., Experimentally based water budgets for dehydrating slabs and consequences for arc magma generation. Earth Planet. Sci. Lett. 163, 361–379 (1998).

[3] S. H. Kirby, E. R. Engdahl, R. Denlinger, “Intermediate depth intraslab earthquakes and arc volcanism as physical expressions of crustal and uppermost mantle metamorphism in subducting slabs”, in *Subduction from Top to Bottom*, G. D. Bebout, D. Scholl, S. Kirby, J. Platt, Eds. (American Geophysical Union, Geophysical Monograph No. 96, 1996), pp. 195–214.

[4] Abers G. A., van Keken P. E., Hacker B. R., The cold and relatively dry nature of mantle forearcs in subduction zones. Nat. Geosci. 10, 333–337 (2017).

[5] Hacker B. R., Peacock S. M., Abers G. A., Holloway S. D., Subduction factory 2. Are intermediate-depth earthquakes in subducting slabs linked to metamorphic dehydration reactions?. J. Geophys. Res. 108, 2030 (2003).

[6] John T., Medvedev S., Rüpke L. H., Andersen T. B., Podladchikov Y. Y., Austrheim H., Generation of intermediate-depth earthquakes by self-localizing thermal runaway. Nat. Geosci. 2, 137–140 (2009).

[7] Kelemen P. B., Hirth G., A periodic shear-heating mechanism for intermediate-depth earthquakes in the mantle. Nature 446, 787–790 (2007). 17429398 10.1038/nature05717

[8] van Keken P. E., Hacker B. R., Syracuse E. M., Abers G. A., Subduction factory: 4. Depth-dependent flux of H_2_O from subducting slabs worldwide. J. Geophys. Res. Solid Earth 116 (2011).

[9] Cerpa N. G., Wada I., Wilson C. R., Fluid migration in the mantle wedge: Influence of mineral grain size and mantle compaction. J. Geophys. Res. Solid Earth 122, 6247–6268 (2017).

[10] Nakajima J., Uchida N., Repeated drainage from megathrusts during episodic slow slip. Nat. Geosci. 11, 351–356 (2018).

[11] Shiina T., Nakajima J., Matsuzawa T., Toyokuni G., Kita S., Depth variations in seismic velocity in the subducting crust: Evidence for fluid-related embrittlement for intermediate-depth earthquakes. Geophys. Res. Lett. 44, 810–817 (2017).

[12] Speranza F., Minelli L., Pignatelli A., Chiappini M., The Ionian Sea: The oldest in situ ocean fragment of the world?. J. Geophys. Res. Solid Earth 117, B12101 (2012).

[13] Pearce F. D., Rondenay S., Sachpazi M., Charalampakis M., Royden L. H., Seismic investigation of the transition from continental to oceanic subduction along the western Hellenic subduction zone. J. Geophys. Res. Solid Earth 117, B07306 (2012).

[14] Shaw B., Jackson J., Earthquake mechanisms and active tectonics of the Hellenic subduction zone. Geophys. J. Int. 181, 966–984 (2010).

[15] Sachpazi M., Laigle M., Charalampakis M., Sakellariou D., Flueh E., Sokos E., Daskalaki E., Galvé A., Petrou P., Hirn A., Slab segmentation controls the interplate slip motion in the SW Hellenic subduction: New insight from the 2008 *M*_*w*_ 6.8 Methoni interplate earthquake. Geophys. Res. Lett. 43, 9619–9626 (2016).

[16] Halpaap F., Rondenay S., Ottemöller L., Seismicity, deformation, and metamorphism in the Western Hellenic subduction zone: New constraints from tomography. J. Geophys. Res. Solid Earth 123, 3000–3026 (2018).

[17] Bostock M. G., The Moho in subduction zones. Tectonophysics 609, 547–557 (2013).

[18] Audet P., Bürgmann R., Possible control of subduction zone slow-earthquake periodicity by silica enrichment. Nature 510, 389–392 (2014). 24943955 10.1038/nature13391

[19] Rontogianni S., Konstantinou K. I., Melis N. S., Evangelidis C. P., Slab stress field in the Hellenic subduction zone as inferred from intermediate-depth earthquakes. Earth Planets Space 63, 139–144 (2011).

[20] Uchida N., Kirby S. H., Okada T., Hino R., Hasegawa A., Supraslab earthquake clusters above the subduction plate boundary offshore Sanriku, northeastern Japan: Seismogenesis in a graveyard of detached seamounts?. J. Geophys. Res. Solid Earth 115, B09308 (2010).

[21] Davey F. J., Ristau J., Fore-arc mantle wedge seismicity under northeast New Zealand. Tectonophysics 509, 272–279 (2011).

[22] Paulatto M., Laigle M., Galve A., Charvis P., Sapin M., Bayrakci G., Evain M., Kopp H., Dehydration of subducting slow-spread oceanic lithosphere in the Lesser Antilles. Nat. Commun. 8, 15980 (2017). 28691714 10.1038/ncomms15980 PMC5508134

[23] Sodoudi F., Brüstle A., Meier T., Kind R., Friederich W.; EGELADOS working group, Receiver function images of the Hellenic subduction zone and comparison to microseismicity. Solid Earth 6, 135–151 (2015).

[24] van Keken P. E., Kita S., Nakajima J., Thermal structure and intermediate-depth seismicity in the Tohoku-Hokkaido subduction zones. Solid Earth 3, 355–364 (2012).

[25] Chuang L., Bostock M., Wech A., Plourde A., Plateau subduction, intraslab seismicity, and the Denali (Alaska) volcanic gap. Geology 45, 647–650 (2017).

[26] Faccenda M., Gerya T. V., Mancktelow N. S., Moresi L., Fluid flow during slab unbending and dehydration: Implications for intermediate-depth seismicity, slab weakening and deep water recycling. Geochem. Geophys. Geosyst. 13, Q01010 (2012).

[27] Morishige M., van Keken P. E., Fluid migration in a subducting viscoelastic slab. Geochem. Geophys. Geosyst. 19, 337–355 (2018).

[28] Ambraseys N. N., Jackson J. A., Seismicity and associated strain of central Greece between 1890 and 1988. Geophys. J. Int. 101, 663–708 (1990).

[29] Papadopoulos G. A., Tsunami hazard in the Eastern Mediterranean: Strong earthquakes and tsunamis in the Corinth Gulf, Central Greece. Nat. Hazards 29, 437–464 (2003).

[30] Wang K., Tréhu A. M., Invited review paper: Some outstanding issues in the study of great megathrust earthquakes—The Cascadia example. J. Geodyn. 98, 1–18 (2016).

[31] Olive J.-A., Pearce F., Rondenay S., Behn M. D., Pronounced zonation of seismic anisotropy in the Western Hellenic subduction zone and its geodynamic significance. Earth Planet. Sci. Lett. 391, 100–109 (2014).

[32] Escartín J., Hirth G., Evans B., Strength of slightly serpentinized peridotites: Implications for the tectonics of oceanic lithosphere. Geology 29, 1023–1026 (2001).

[33] National Observatory of Athens, Database of revised events (2018); http://bbnet.gein.noa.gr/HL/databases/database.

[34] Chamberlain C. J., Hopp C. J., Boese C. M., Warren-Smith E., Chambers D., Chu S. X., Michailos K., Townend J., EQcorrscan: Repeating and near-repeating earthquake detection and analysis in Python. Seismol. Res. Lett. 89, 173–181 (2017).

[35] Waldhauser F., Ellsworth W. L., A double-difference earthquake location algorithm: Method and application to the northern Hayward fault, California. Bull. Seismol. Soc. Am. 90, 1353–1368 (2000).

[36] Havskov J., Ottemoller L., SeisAn Earthquake Analysis Software. Seismol. Res. Lett. 70, 532–534 (1999).

[37] Lienert B. R., Havskov J., A computer program for locating earthquakes both locally and globally. Seismol. Res. Lett. 66, 26–36 (1995).

[38] Bleibinhaus F., Gebrande H., Crustal structure of the Eastern Alps along the TRANSALP profile from wide-angle seismic tomography. Tectonophysics 414, 51–69 (2006).

[39] Beyreuther M., Barsch R., Krischer L., Megies T., Behr Y., Wassermann J., ObsPy: A Python toolbox for seismology. Seismol. Res. Lett. 81, 530–533 (2010).

[40] Tichelaar B. W., Ruff L. J., How good are our best models? Jackknifing, bootstrapping, and earthquake depth. Eos 70, 593–606 (1989).

[41] Snoke J. A., Munsey J. W., Teague A. G., Bollinger G. A., A program for focal mechanism determination by combined use of polarity and SV-P amplitude ratio data. Earthquake Notes 55, 15 (1984).

[42] Perrin A., Goes S., Prytulak J., Davies D. R., Wilson C., Kramer S., Reconciling mantle wedge thermal structure with arc lava thermobarometric determinations in oceanic subduction zones. Geochem. Geophys. Geosyst. 17, 4105–4127 (2016).

[43] Davies D. R., Wilson C. R., Kramer S. C., Fluidity: A fully unstructured anisotropic adaptive mesh computational modeling framework for geodynamics. Geochem. Geophys. Geosyst. 12, Q06001 (2011).

[44] Zhu H., Bozdağ E., Tromp J., Seismic structure of the European upper mantle based on adjoint tomography. Geophys. J. Int. 201, 18–52 (2015).

[45] Syracuse E. M., van Keken P. E., Abers G. A., The global range of subduction zone thermal models. Phys. Earth Planet. Inter. 183, 73–90 (2010).

[46] Holland T. J. B., Powell R., An internally consistent thermodynamic data set for phases of petrological interest. J. Metamorph. Geol. 16, 309–343 (1998).

[47] Connolly J. A. D., Computation of phase equilibria by linear programming: A tool for geodynamic modeling and its application to subduction zone decarbonation. Earth Planet. Sci. Lett. 236, 524–541 (2005).

[48] Hacker B. R., Abers G. A., Subduction Factory 3: An excel worksheet and macro for calculating the densities, seismic wave speeds, and H_2_O contents of minerals and rocks at pressure and temperature. Geochem. Geophys. Geosyst. 5, Q01005 (2004).

[49] Xu W., Lithgow-Bertelloni C., Stixrude L., Ritsema J., The effect of bulk composition and temperature on mantle seismic structure. Earth Planet. Sci. Lett. 275, 70–79 (2008).

[50] Eeken T., Goes S., Pedersen H. A., Arndt N. T., Bouilhol P., Seismic evidence for depth-dependent metasomatism in cratons. Earth Planet. Sci. Lett. 491, 148–159 (2018).

[51] Abers G. A., Hacker B. R., A MATLAB toolbox and excel workbook for calculating the densities, seismic wave speeds, and major element composition of minerals and rocks at pressure and temperature. Geochem. Geophys. Geosyst. 17, 616–624 (2016).

[52] Goes S., Armitage J., Harmon N., Smith H., Huismans R., Low seismic velocities below mid-ocean ridges: Attenuation versus melt retention. J. Geophys. Res. 117, B12403 (2012).

[53] G. Laske, A. Dziewonski, G. Masters, Reference Earth Model (2013); https://igppweb.ucsd.edu/~gabi/rem.html.

[54] Nadeau R. M., Johnson L. R., Seismological studies at Parkfield VI: Moment release rates and estimates of source parameters for small repeating earthquakes. Bull. Seismol. Soc. Am. 88, 790–814 (1998).

[55] Uchida N., Iinuma T., Nadeau R. M., Bürgmann R., Hino R., Periodic slow slip triggers megathrust zone earthquakes in northeastern Japan. Science 351, 488–492 (2016). 26823425 10.1126/science.aad3108

[56] Hanks T. C., Kanamori H., A moment magnitude scale. J. Geophys. Res. Solid Earth 84, 2348–2350 (1979).

[57] Scordilis E. M., Kementzetzidou D., Papazachos B. C., Local magnitude calibration of the Hellenic unified seismic network. J. Seismol. 20, 319–332 (2016).

[58] Makropoulos K., Kaviris G., Kouskouna V., An updated and extended earthquake catalogue for Greece and adjacent areas since 1900. Nat. Hazards Earth Syst. Sci. 12, 1425–1430 (2012).

[59] Bocchini G. M., Brüstle A., Becker D., Meier T., van Keken P. E., Ruscic M., Papadopoulos G. A., Rische M., Friederich W., Tearing, segmentation, and backstepping of subduction in the Aegean: New insights from seismicity. Tectonophysics 734–735, 96–118 (2018).

[60] Reyners M., Eberhart-Phillips D., Stuart G., Nishimura Y., Imaging subduction from the trench to 300 km depth beneath the central North Island, New Zealand, with *Vp* and *Vp/Vs*. Geophys. J. Int. 165, 565–583 (2006).

[61] Laigle M., Hirn A., Sapin M., Bécel A., Charvis P., Flueh E., Diaz J., Lebrun J.-F., Gesret A., Raffaele R., Galvé A., Evain M., Ruiz M., Kopp H., Bayrakci G., Weinzierl W., Hello Y., Lépine J.-C., Viodé J.-P., Sachpazi M., Gallart J., Kissling E., Nicolich R., Seismic structure and activity of the north-central Lesser Antilles subduction zone from an integrated approach: Similarities with the Tohoku forearc. Tectonophysics 603, 1–20 (2013).

[62] Wilson C. R., Spiegelman M., van Keken P. E., Hacker B. R., Fluid flow in subduction zones: The role of solid rheology and compaction pressure. Earth Planet. Sci. Lett. 401, 261–274 (2014).

[63] Omlin S., Malvoisin B., Podladchikov Y. Y., Pore fluid extraction by reactive solitary waves in 3-D. Geophys. Res. Lett. 44, 9267–9275 (2017).

[64] Chakraborty S., A new mechanism for upper crustal fluid flow driven by solitary porosity waves in rigid reactive media?. Geophys. Res. Lett. 44, 10324–10327 (2017).

[65] Connolly J. A. D., Podladchikov Y. Y., An analytical solution for solitary porosity waves: Dynamic permeability and fluidization of nonlinear viscous and viscoplastic rock. Geofluids 15, 269–292 (2015).

[66] G. Thompson, C. Reyes, GISMO—A seismic data analysis toolbox for MATLAB (2017).

[67] Tzanis A., Efstathiou A., Chailas S., Stamatakis M., Evidence of recent plutonic magmatism beneath Northeast Peloponnesus (Greece) and its relationship to regional tectonics. Geophys. J. Int. 212, 1600–1626 (2018).

[68] Galanopoulos D., Sakkas V., Kosmatos D., Lagios E., Geoelectric investigation of the Hellenic subduction zone using long period magnetotelluric data. Tectonophysics 409, 73–84 (2005).

[69] S. Rondenay, Multi-disciplinary experiments for dynamic understanding of subduction under the Aegean Sea. International Federation of Digital Seismograph Networks. Other/Seismic Network (2006); 10.7914/SN/XS_2006.

[70] W. Friederich, T. Meier, Egelados project 2005/07. International Federation of Digital Seismograph Networks. Other/Seismic Network (2005); 10.14470/M87550267382.

[71] A. Paul, H. Karabulut; RESIF, Seismic network XY:SIMBAAD temporary experiment—Backbone of broadband stations. RESIF - Réseau Sismologique et géodésique Français. International Federation of Digital Seismograph Networks. Other/Seismic Network (2013); 10.15778/resif.xy2007.

[72] Corinth Rift Laboratory Team And RESIF Datacenter, CL—Corinth Rift Laboratory Seismological Network (CRLNET). International Federation of Digital Seismograph Networks. Other/Seismic Network (2013); 10.15778/resif.cl.

[73] University of Athens, University of Athens Seismological Laboratory. International Federation of Digital Seismograph Networks. Other/Seismic Network (2008); 10.7914/SN/HA.

[74] National Observatory of Athens, Institute of Geodynamics, National Observatory of Athens Seismic Network. International Federation of Digital Seismograph Networks. Other/Seismic Network (1997); 10.7914/SN/HL.

[75] University of Patras; Geology Department; Seismological Laboratory, PSLNET, permanent seismic network operated by the University of Patras, Greece. International Federation of Digital Seismograph Networks. Other/Seismic Network (2000); 10.7914/SN/HP.

[76] Aristotle University of Thessaloniki, Seismological Network, Permanent Regional Seismological Network operated by the Aristotle University of Thessaloniki. International Federation of Digital Seismograph Networks. Other/Seismic Network (1981); 10.7914/SN/HT.

[77] GEOFON Data Centre, GEOFON Seismic Network (1993). Deutsches GeoForschungsZentrum GFZ; 10.14470/tr560404.

[78] Konstantinou K. I., Melis N. S., Boukouras K., Routine regional moment tensor inversion for earthquakes in the Greek region: The national observatory of Athens (NOA) database (2001–2006). Seismol. Res. Lett. 81, 750–760 (2010).

[79] Serpetsidaki A., Sokos E., Tselentis G.-A., A ten year moment tensor database for Western Greece. Phys. Chem. Earth 95, 2–9 (2016).

[80] L. Ottemöller, P. Voss, J. Havskov, Seisan earthquake analysis software for Windows, Solaris, Linux and MacOSX (2016); http://seis.geus.net/software/seisan/seisan.pdf.

[81] Schorlemmer D., Euchner F., Kästli P., Saul J., QuakeML: Status of the XML-based seismological data exchange format. Ann. Geophys. 54, 1 (2011).

