# Supplementary material for: Earthquakes track subduction fluids from slab source to mantle wedge sink
Source: Sci Adv. 2019 Apr 3;5(4):eaav7369. doi: 10.1126/sciadv.aav7369 (PMC6447373; doi:10.1126/sciadv.aav7369)
Supplement: http://advances.sciencemag.org/cgi/content/full/5/4/eaav7369/DC1 [file supp_5_4_eaav7369__index.html]

Science Advances | Science Advances

## Supplementary Materials

**This PDF file includes:**

- Supplementary Text
- Mantle wedge seismicity in other subduction zones
- Episodicity of mantle wedge earthquakes and velocity of fluid migration
- Fig. S1. Along-trench profiles of mantle wedge seismicity and P-velocity structure, plotted as seen from the trench.
- Fig. S2. Temporal evolution of seismicity in the Tripoli cluster.
- Fig. S3. P-velocity to S-velocity (*V*p/*V*s) ratio structure beneath western Greece.
- Fig. S4. Focal mechanisms of the Tripoli cluster.
- Fig. S5. Estimates of completeness magnitude, *M*c, for various catalogs of deep earthquakes (>35 km) below western Greece.
- Fig. S6. Comparison of seismic images and calculated seismic structure along the cross section of Fig. 1D.
- Fig. S7. Hypocenters displayed with their relative location errors.
- Fig. S8. Earthquake distribution and electric resistivity structure below the Peloponnese.
- Table S1. Seismograph networks from western Greece used in the waveform processing.
- Table S2. Focal mechanism solutions of deep earthquakes in the Western Hellenic subduction zone.
- Table S3. Locations of mantle wedge seismicity displayed in cross sections in Fig. 4.
- External Data file S1. Deep earthquake hypocenters in Greece.
- External Data file S2. Deep earthquake focal mechanisms in Greece.
- External Data file S3. Earthquake arrival time picks.
- External Data file S4. Model of the subduction plate interface.
- External Data file S5. Thermal structure model of the subduction zone.
- References (*59*–*81*)

Download PDF

**Files in this Data Supplement:**

- Adobe PDF - aav7369\_SM.pdf
