# Supplementary material for: Earthquakes track subduction fluids from slab source to mantle wedge sink
Source: Sci Adv. 2019 Apr 3;5(4):eaav7369. doi: 10.1126/sciadv.aav7369 (PMC6447373; doi:10.1126/sciadv.aav7369)
Supplement: Download PDF [file aav7369_SM.pdf]

Science Advances
AAAS

advances.sciencemag.org/cgi/content/full/5/4/eaav7369/DC1

Supplementary Materials for

Earthquakes track subduction fluids from slab source to mantle wedge sink

Felix Halpaap*, Stéphane Rondenay, Alexander Perrin, Saskia Goes, Lars Ottemöller, Håkon Austrheim, Robert Shaw, Thomas Eeken

*Corresponding author. Email: felix.halpaap@uib.no

Published 3 April 2019, *Sci. Adv.* **5**, eaav7369 (2019)
DOI: 10.1126/sciadv.aav7369

This PDF file includes:

Supplementary Text
Mantle wedge seismicity in other subduction zones
Episodicity of mantle wedge earthquakes and velocity of fluid migration
Fig. S1. Along-trench profiles of mantle wedge seismicity and P-velocity structure, plotted as seen from the trench.
Fig. S2. Temporal evolution of seismicity in the Tripoli cluster.
Fig. S3. P-velocity to S-velocity (V_p/V_s) ratio structure beneath western Greece.
Fig. S4. Focal mechanisms of the Tripoli cluster.
Fig. S5. Estimates of completeness magnitude, M_c, for various catalogs of deep earthquakes (>35 km) below western Greece.
Fig. S6. Comparison of seismic images and calculated seismic structure along the cross section of Fig. 1D.
Fig. S7. Hypocenters displayed with their relative location errors.
Fig. S8. Earthquake distribution and electric resistivity structure below the Peloponnese.
Table S1. Seismograph networks from western Greece used in the waveform processing.
Table S2. Focal mechanism solutions of deep earthquakes in the Western Hellenic subduction zone.
Table S3. Locations of mantle wedge seismicity displayed in cross sections in Fig. 4.
External Data file S1. Deep earthquake hypocenters in Greece.
External Data file S2. Deep earthquake focal mechanisms in Greece.
External Data file S3. Earthquake arrival time picks.
External Data file S4. Model of the subduction plate interface.
External Data file S5. Thermal structure model of the subduction zone.
References (*59*–*81*)

Supplementary Text

Mantle wedge seismicity in other subduction zones

Figure 4 shows the worldwide locations where clusters of mantle wedge seismicity have been observed. Cross sections from these locations reveal how the Tripoli cluster (A) compares to selected clusters offshore Sanriku (Japan, Fig. 4 B), eastern Crete (Greece, C), below Raukumara (New Zealand, D), and below Martinique (lesser Antilles, E). All occurrences appear in cold subduction zones with generally dry mantle wedge corners (see map in Fig. 4). Here, we briefly describe each region.

Cross section A, through the Tripoli cluster, shows the deep earthquakes depicted in Fig. 3 of the main text. In addition, it includes shallow earthquake hypocenters published by NOA (2011 – 2017, (*33*)) that fulfill the following criteria: location errors smaller than 3 km, minimum number of 12 phase picks or 8 observing stations, and azimuthal gap of less than 180º.

Cross section B, eastern Crete, comprises earthquakes from two sources: i) the NOA catalog, and ii) the catalog from regional studies in refs. (*23*, *59*). We select NOA hypocenters with the same criteria as in cross section A, except that we plot hypocenters from all depths and loosen the gap criterion to 225º for earthquakes observed with more than 18 picks or 12 stations, to include offshore earthquakes south of Crete. The second catalog is based on multiple dense temporary seismic experiments on- and offshore Crete. These were deployed to study microseismic activity of the subduction zone where permanent monitoring is hampered by sparse land station coverage. With these temporary networks, Sodoudi et al. (*23*) imaged the plate interface and Moho discontinuities in the region with PS-receiver function analysis. The location of the plate interface and hypocenters in Fig. 4 B indicate two regions of high seismicity within subducting crust: a shallow segment updip from the ~35 km interface depth, and a deep segment downdip from the ~50 km interface depth. The two segments are separated by a ~60 km wide region of reduced seismicity. At the upper tip of the deep segment, earthquake activity appears to stretch into the mantle wedge and connect to seismicity clusters in the lower part of the overriding crust. The high-low-high seismicity pattern does not affect seismicity within the subducting mantle (> 10 km below the interface), which extends continuously below the crustal gap.

Cross section C, offshore Sanriku, shows hypocenters relocated by double-difference inversion in ref. (*20*). Seismic discontinuities in the region are constrained by P-S conversions from deep local earthquakes (*20*). The upper and lower seismic plane of the WBZ dipping westward are clearly separated. A dense cluster of seismicity clearly exists on the interface at 45 km depth, above which a large region of seismic activity stretches into the trenchward part of the mantle wedge. A ~20 km wide gap in crustal seismicity is present ~20 km updip from the interface cluster, but some earthquakes occur right updip from the interface cluster. We note that this region falls right above the shallow limit of the lower seismicity plane, where some hypocenters appear to from a link between the lower and upper planes. This may reflect the existence of a vertical fluid pathway from the lower to the upper seismic zone.

Cross section D, at Raukumara, shows mantle wedge seismicity analyzed in ref. (*21*). Hypocenters were obtained by relocation in a 3-D velocity model (*60*), with plate interface and Moho discontinuities constrained by seismicity, local earthquake arrival time tomography, and a seismic reflection image. The cross section clearly shows an intense

cluster of seismicity extending ~15 km upward from a ~40 km thick WBZ dipping to the west. WBZ seismicity stretches from the interface well into the subducting mantle. Updip of the mantle wedge cluster, there are hardly any earthquakes in a ~30 km wide section of subducting crust. This seismicity gap only exists in the subducting crust (as in Crete), with earthquake activity appearing uninterrupted in the subducting mantle below. In the context of our proposed fluid migration model, this dichotomy suggests two clearly separated migration pathways in the subducting crust and mantle.

Cross section E, across Martinique, shows seismicity that was first analyzed in ref. (*61*) and relocated in a 3-D velocity model in ref. (*22*). The plate interface and Moho discontinuities are from ref. (*22*) and were constrained by seismicity, local earthquake arrival time tomography, and seismic reflection imaging (*22*, *61*). A dense cluster of seismicity appears on the interface at ~45 km depth and is overlain by a region of distributed mantle wedge seismicity. It is not immediately clear why the mantle wedge seismicity is more diffuse than in other regions. But it appears that interface seismicity extends over a larger depth segment here. This could indicate that venting of fluids occurs over a broader region below Martinique. Updip from the main cluster of interface seismicity (~45 km depth), the subducting crust is nearly aseismic.

Episodicity of mantle wedge earthquakes and velocity of fluid migration

Figure S1 shows the occurrence in time of earthquakes in the Tripoli cluster, with a distinction between earthquakes in the slab, on the interface and in the mantle wedge. These earthquakes, unlike their counterparts in Japan (*10*), do not appear to show any time-dependent behavior. To investigate whether this discrepancy is due to a difference in subduction rate, we turn to recent studies that have researched the mechanisms by which fluids produced by dehydration reactions flow through the slab and into the mantle wedge. The mechanisms are debated, but a strong candidate is compaction-driven flow in the form of porosity waves (*9*, *27*, *62*).

Even though the two-phase flow models currently used are strictly only valid for the ductile domain of (hot) mantle wedge and lower crust, recent theoretical studies suggest that reaction-induced compaction changes may lead to analogous porosity waves in the brittle-elastic domain (*63*, *64*). These findings are important, because if we can assume that fluid is transported by porosity waves, then we can evaluate how a difference in subduction rate may affect the velocity at which fluid migrates through the system.

Here, we assume that compaction-driven flow can occur in the subducting crust and estimate how the fluid velocity varies as a function of subduction rate. First, we can establish that an increase in subduction rate linearly affects the fluid production rate, as slab rocks pass the dehydration window in a shorter time span. Second, since the formation of porosity is caused by the production of fluids (*65*), we can establish that porosity is also linearly related to subduction rate. Third, based on the knowledge that, at low porosities, permeability is approximately related to the cube of porosity (eq. 17 in (*65*)), we can establish that permeability is related to the cube of the subduction rate. Lastly, as the fluid velocity is equal to the ratio of permeability to porosity, we find that fluid velocity is proportional to the square of the subduction rate.
We can now compare fluid migration rates between NE Japan with western Greece. In NE Japan, where the subduction velocity is 82.7 mm/yr (*8*), mantle wedge seismicity has been shown to occur episodically with a 1-year cycle. The subduction velocity is about 2.4 times higher than in western Greece (35 mm/yr (*13*)), where we have not detected any episodicity. A 2.4-fold slower subduction velocity in western Greece would result in a fluid migration

velocity that is considerably smaller, i.e., by a factor of $2.4^2 \approx 6$, than in NE Japan. With an expected cycle of 6 years, this episodicity would be very difficult to detect robustly in Greece yet, where our high-resolution data are limited to a time span of 11 years.

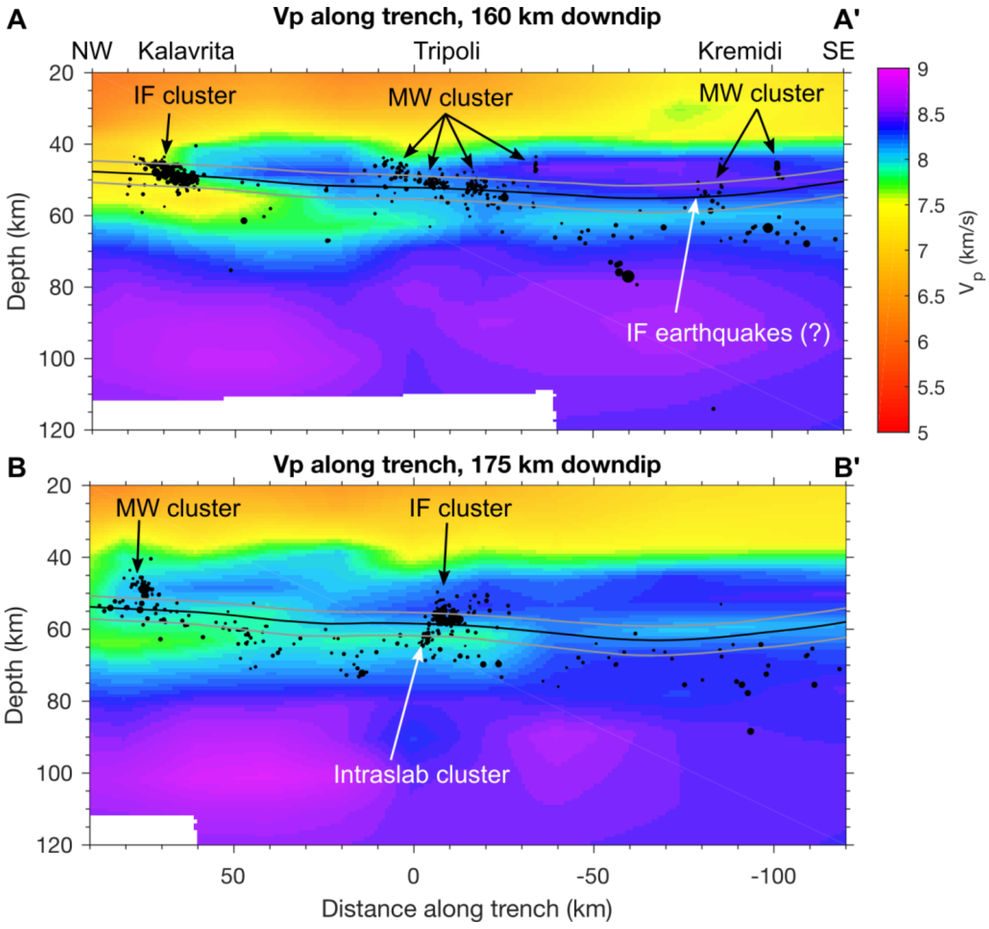

Fig. S1. Along-trench profiles of mantle wedge seismicity and P-velocity structure, plotted as seen from the trench. The two profiles crosscut those shown in Fig. 2 B-E orthogonally at an along-dip distance of 70 km (A, corresponding to cross section A-A' in Fig. 2) and 90 km (B). Earthquakes with absolute or relative location errors < 5 km and up to ±10 km away from each cross section are shown. Since the plate interface dips downward away from the viewer in both cross-sections, it is marked with a black line and two gray lines which represent the central, uppermost and lowermost positions of the interface. Both the Kalavrita and Tripoli clusters comprise a single, highly-active patch of earthquakes on the interface (IF, see A and B, respectively). Mantle wedge earthquakes (MW) below Kalavrita and Kremidi cluster along one main subvertical feature. In contrast, below Tripoli, they form four subclusters that branch out from the interface patch in different directions (A). These observations suggest that fluids generally escape the slab through highly localized interface vents, but once in the mantle wedge they can sometimes follow multiple pathways upwards.

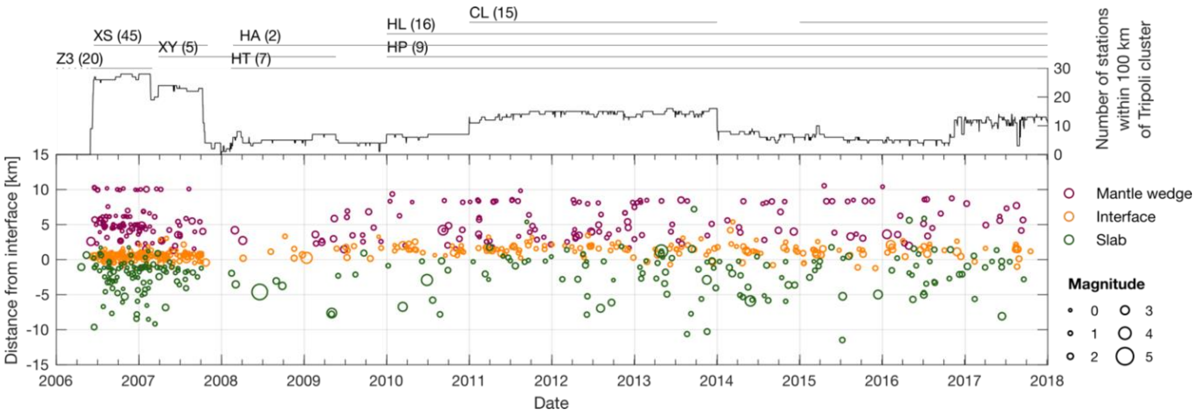

Fig. S2. Temporal evolution of seismicity in the Tripoli cluster. The curve on top depicts the number of stations within 100 km radius for which we could download and process waveform data. The apparent periods with more (2006-06 to 2007-10) or less (2007-11 to 2009-02) seismic activity are thus due to differences in network coverage. Hence we detect no apparent cyclic behavior of earthquakes in the mantle wedge (magenta circles), compared to the annual cyclicity observed in Japan (*10*).

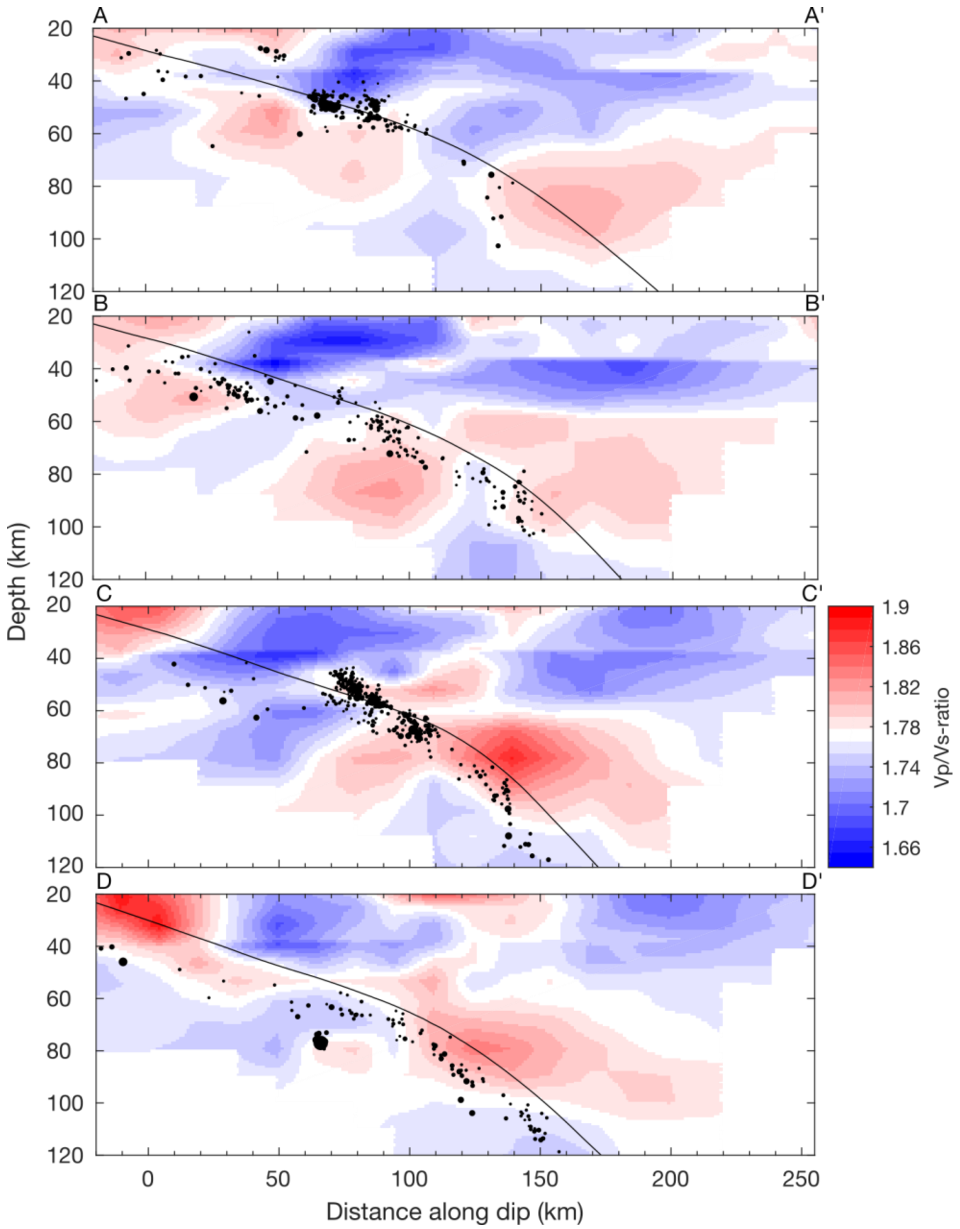

Fig. S3. P-velocity to S-velocity (V_p/V_s) ratio structure beneath western Greece. (**A** – **D**) Cross sections are in the same locations as in Fig. 2; the tomographic model is from Halpaap et al. (*16*). Note the areas of high Vp/Vs-ratio (>1.8) in the mantle wedge above the inferred locus of blueschist-dehydration at 80 – 140 km depth, likely indicating the release of fluid and formation of subarc melt. The low Vp/Vs-ratio (< 1.7) in the lower overriding crust, close to the interface, likely indicates quartz-enriched rocks, where dehydration fluids traveling updip were able to precipitate SiO_2. Within the cluster of interface and mantle wedge seismicity, we observe a small region (10 km thick) of high Vp/Vs-ratio consistent with the proposed fluid channeling at that location. Even though this feature is at the limit of spatial resolution for the tomographic model, it agrees well with a low-resistivity anomaly imaged in a geoelectric profile at that same location (fig. S8).

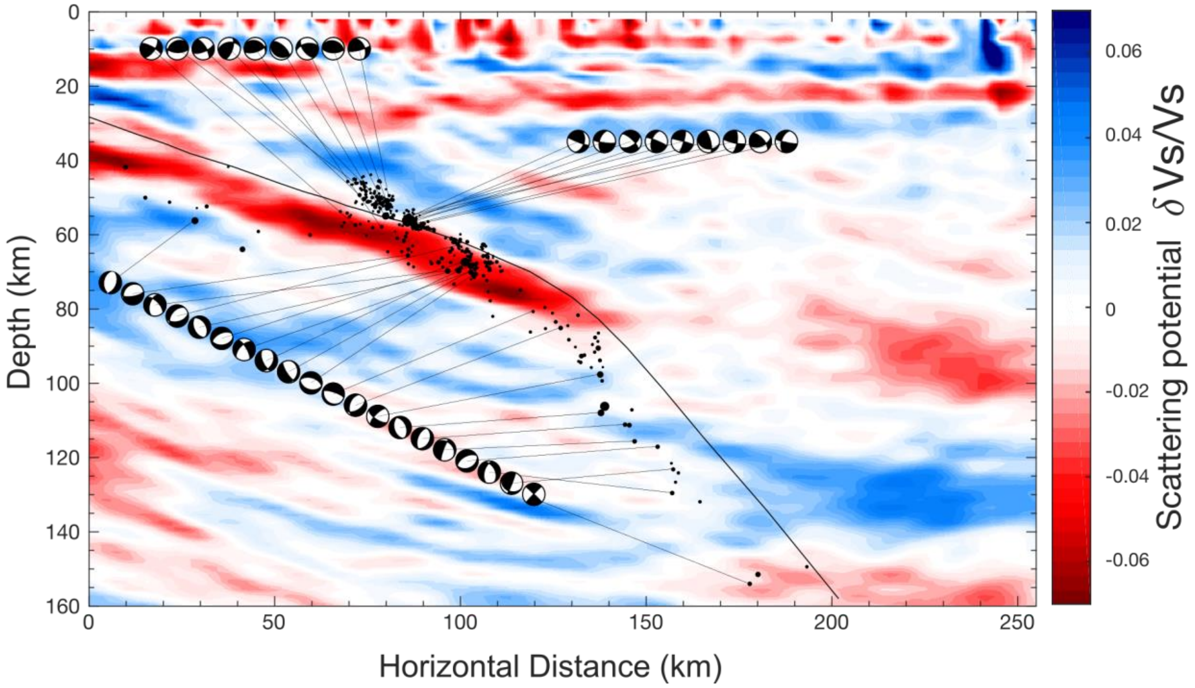

Fig. S4. Focal mechanisms of the Tripoli cluster. The cross section is equivalent to that of Fig. 3 A in the main text, but identifies the individual earthquakes for which we calculated focal mechanism solutions. The three sets of beach balls are grouped according to the earthquake location, clockwise from the top: mantle wedge earthquakes; interface earth–quakes; and intraslab earthquakes.

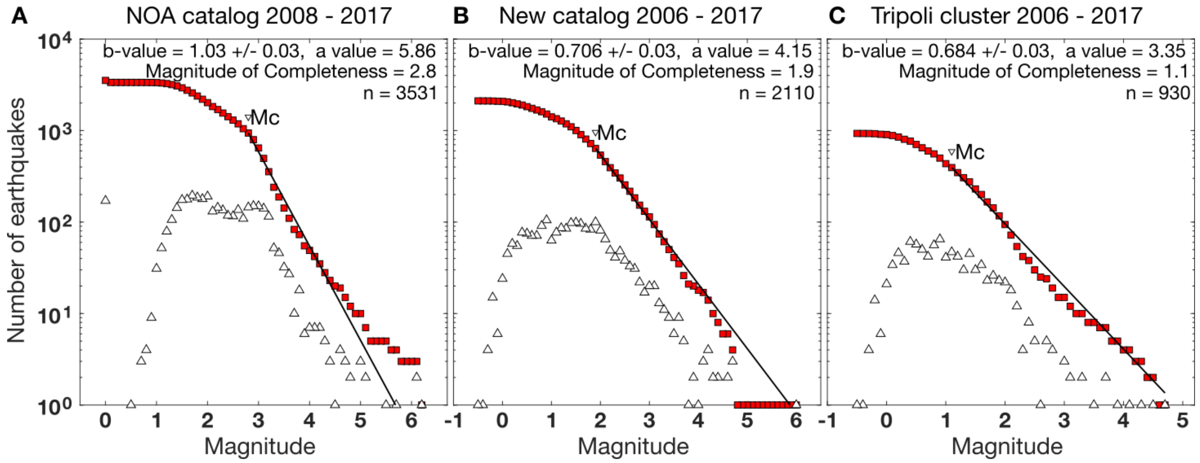

Fig. S5. Estimates of completeness magnitude, M_c, for various catalogs of deep earthquakes (>35 km) below western Greece. Frequency magnitude distribution is indicated by red squares (cumulative number of earthquakes greater than a certain magnitude) and white triangles (incremental number of earthquakes). Completeness magnitudes are determined from a 90 %-maximum likelihood solution fit in the Gismo seismic data analysis toolbox (*66*). (**A**) The NOA catalog contains the largest number of events, but many of these may have occurred in reality at shallow depth due to large locations errors – especially in the vertical direction. (**B**) The "New catalog" is the regional dataset assembled as part of this study, excluding 62 events that occurred prior to June 2006 (prior to the start of the Medusa deployment) as these were recorded at a time when station coverage was insufficient to detect small magnitude earthquakes. (**C**) The "Tripoli cluster" catalog contains only events from the mantle wedge, interface and intraslab subclusters below Tripoli. (see Fig. 3 D).

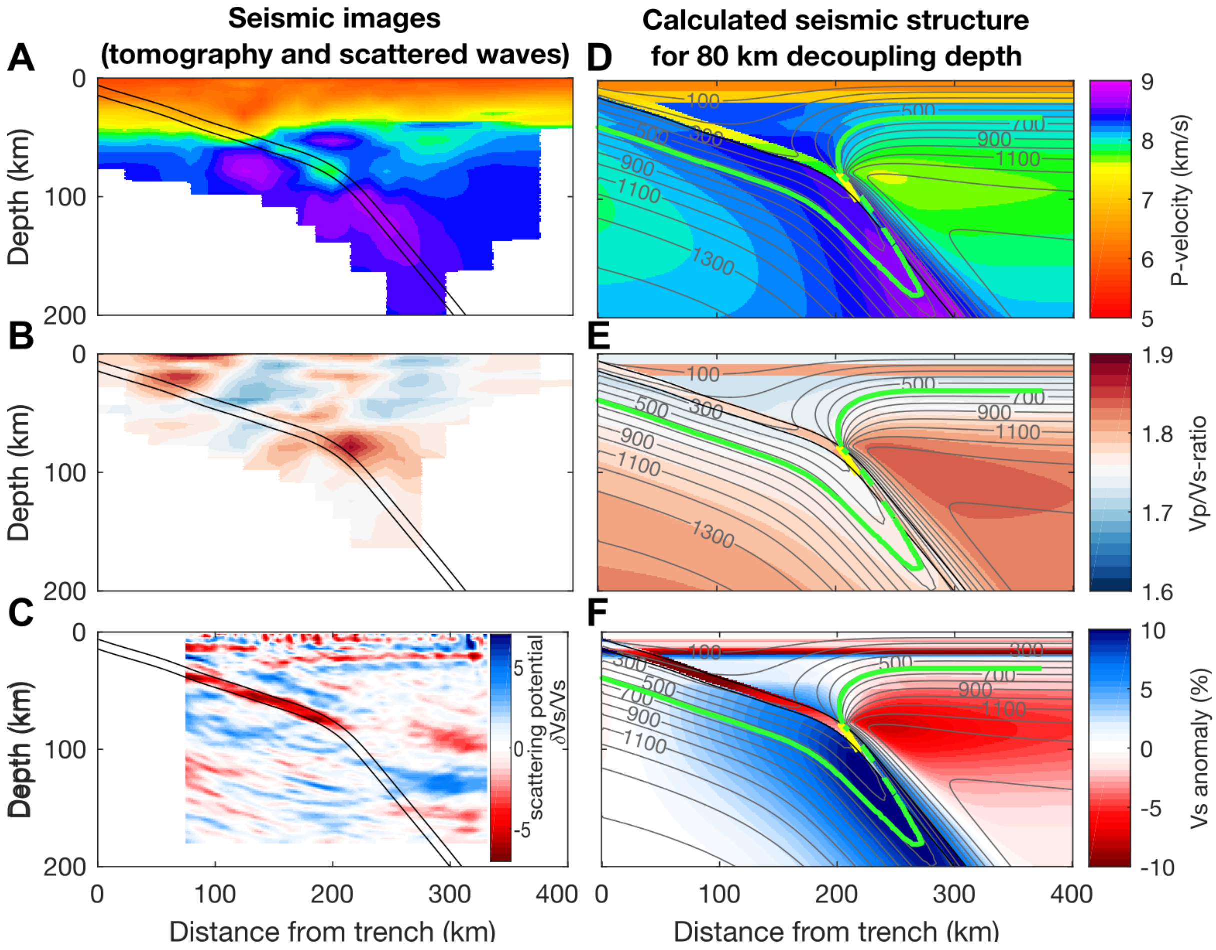

Fig. S6. Comparison of seismic images and calculated seismic structure along the cross section of Fig. 1D. (**A** – **C**) Seismic images from tomography (**A** P-velocity, **B** Vp/Vs-ratio) and from scattered teleseismic wave imaging (**C** relative S-wavespeed perturbations $\delta\beta/\beta$). (**D** – **F**) Calculated seismic structure in terms of **D** P-velocity, **E** Vp/Vs-ratio, **F** S-wavespeed anomaly in comparison to an average 1-D velocity model. Relative S-wavespeed perturbations and S-wavespeed anomalies are not equivalent as the former depends also on the sharpness of a contrast. Still, the S-wavespeed anomalies visualize where sharp wavespeed contrasts exist, in particular at the top of the subducting low-velocity crust. Grey contour lines indicate isotemperatures in °C. Our phase stability calculations include a wide variety of hydrous minerals in the slab (see text), but here we only show the two main water-releasing reactions: antigorite-out in the mantle (indicated by green lines), and blueschist-to-eclogite in the subducted crust (indicated by yellow lines). Note that the imaged Vp/Vs-ratios appear to be influenced primarily by the concentration of fluids near the depths at which the slab dehydrates – something that cannot be fully reproduced in the forward modeling of seismic velocities, as the modeling assumes a dry upper plate and a homogenous hydration in the mantle wedge.

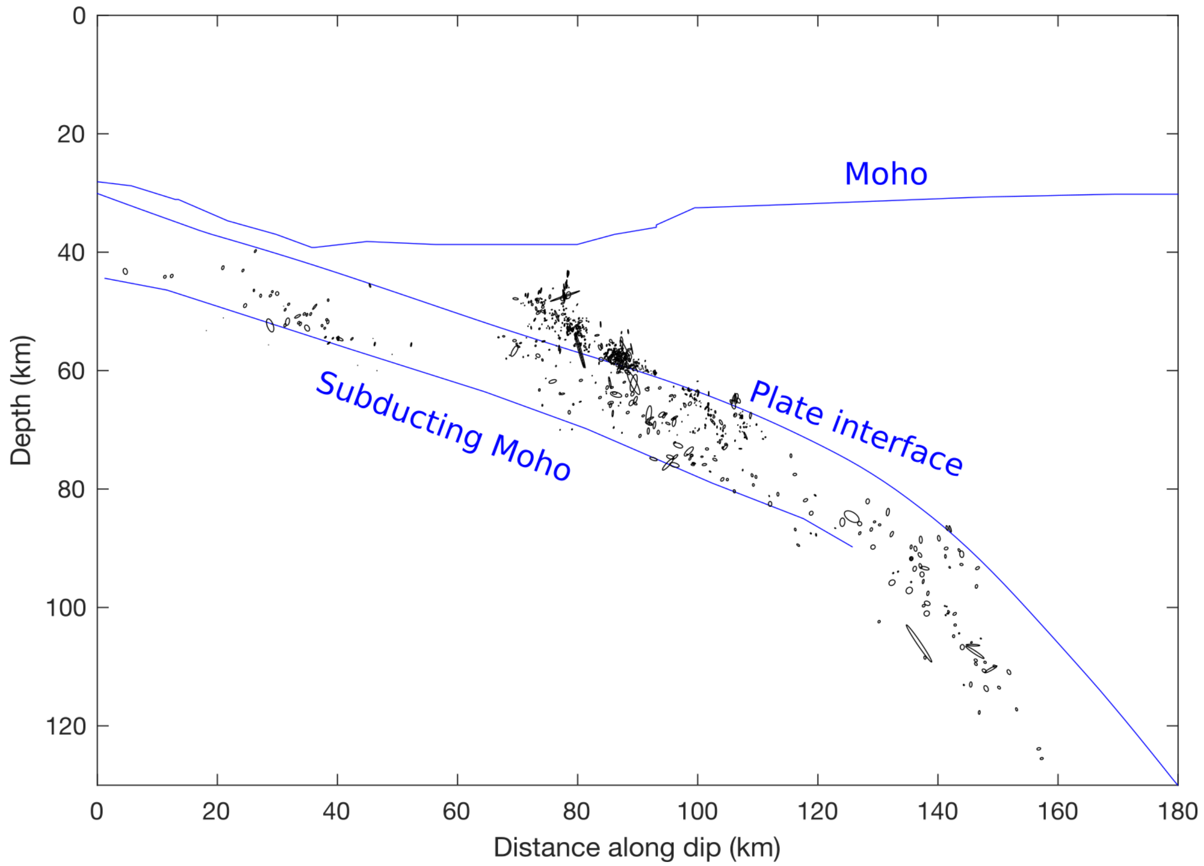

Fig. S7. Hypocenters displayed with their relative location errors. These errors were estimated from n=1000 inversions of jackknife-resampled differential arrival time data. Errors are on average 0.19 km in the horizontal direction [0.20 km in the dip direction (x), 0.17 km in the along-strike direction (y)], and 0.21 km in the vertical direction. The variations in error from one event to another are caused primarily by variations in station coverage at time when the events were recorded.

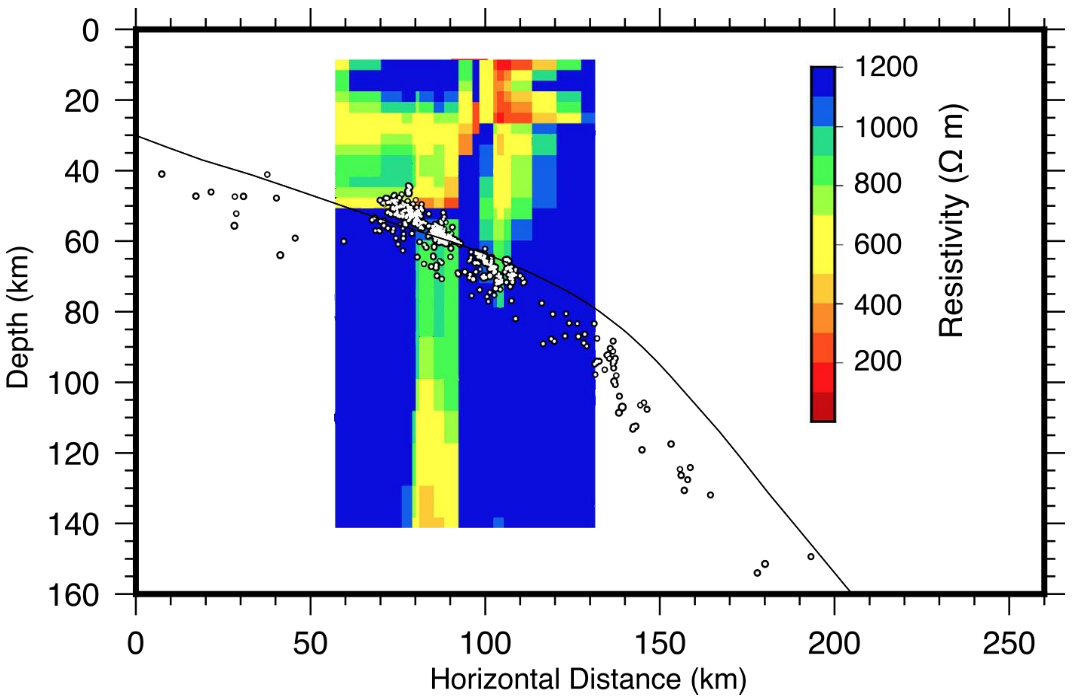

Fig. S8. Earthquake distribution and electric resistivity structure below the Peloponnese. We superimpose the earthquakes from the profile in Fig. 3 A (main text) onto the resistivity image from Tzanis et al. (*67*), which stems from a magnetotelluric survey of the Peloponnese that was first reported in Galanopoulos et al. (*68*). The magnetotelluric profile ran parallel to the scattered wave image shown in Fig. 3 A, about 20 km farther southeast. A vertically stretched low-resistivity anomaly coincides precisely with the cluster of interface seismicity, suggesting the presence of highly conductive material, e.g. saline slab fluid at that location. Vertically, the anomaly is likely stretched because depth tends to be poorly constrained in such magnetotelluric images.

Table S1. Seismograph networks from western Greece used in the waveform processing. Here we indicate the time periods that we used for the processing of waveform data from each network. We only used data that were publicly available through data services of the Federation of Digital Seismograph Networks at the time of the study.

Project / Institution	Network	Time period	Number of stations	Reference
Medusa	XS, temporary	2006-06 to 2007-10	46	(*69*)
Egelados	Z3, temporary	2006-06 to 2007-04	20	(*70*)
Simbaad	XY, temporary	2007 to 2009	5	(*71*)
Corinth Rift Laboratory	CL, permanent	2011 to 2013 and 2016 to 2017	15	(*72*)
University of Athens	HA, permanent	2008 to 2017	2	(*73*)
National Observatory of Athens (NOA)	HL, permanent	2010 to 2017	16	(*74*)
University of Patras	HP, permanent	2011 to 2017	9	(*75*)
University of Thessaloniki	HT, permanent	2008 to 2017	7	(*76*)
Geofon, Deutsches Geoforschungszentrum	GE, permanent	2006 to 2017	3	(*77*)

Table S2. Focal mechanism solutions of deep earthquakes in the Western Hellenic subduction zone. For solutions from the literature, moment tensor solutions are available in addition to the solutions shown here. The reference to "t.P." refers to solutions recalculated in this paper. For the solutions from the NOA database (*33*), the routine calculations method is described in ref. (*78*). This list is also available in plain text format in external database S2 (see link in caption below).

Date, time	Latitude	Longitude	Depth (km)	Strike	Dip	Rake	Reference
2006-01-08T11:34:54.200	36.14367	23.42219	62.791	195.0	42.0	53.0	(*14*)
2006-06-13T02:25:59.620	37.82292	21.89912	56.045	252.8	33.9	32.8	t.P., (*33*)
2006-06-15T21:47:45.620	37.58525	22.64383	57.545	111.8	76.0	87.9	t.P.
2006-06-19T11:31:50.070	37.61347	22.53638	49.479	13.2	72.0	-87.9	t.P.
2006-07-03T21:27:32.160	38.11814	22.00875	50.713	356.8	87.1	-73.0	t.P.
2006-07-05T23:02:17.820	37.80508	21.98657	54.259	289.8	40.0	50.7	t.P.
2006-07-14T09:57:14.500	37.20080	22.15691	56.238	194.1	74.5	4.0	t.P., (*33*)
2006-08-28T04:22:46.660	37.35656	22.60315	57.402	180.0	58.0	-90.0	t.P.
2006-09-07T23:51:22.920	37.60800	22.62542	57.270	210.6	40.1	-68.0	t.P.
2006-09-25T04:49:11.530	37.85903	22.59062	70.850	188.0	10.0	-90.0	t.P.
2006-10-11T17:30:59.120	37.51822	22.59631	53.003	295.0	58.3	-66.3	t.P.
2006-10-29T23:16:10.560	37.70723	22.76923	69.257	218.6	84.7	-31.6	t.P.
2006-11-20T21:02:28.920	37.59386	22.51497	50.423	68.5	48.1	-23.6	t.P.
2006-12-03T22:23:13.240	37.46497	22.61664	57.883	46.1	62.3	-81.0	t.P.
2007-01-10T08:16:08.650	37.71160	22.70829	62.932	224.8	43.4	43.3	t.P.
2007-02-07T21:19:13.280	38.11929	22.00810	50.791	204.7	46.0	87.2	t.P.
2007-02-09T07:09:35.650	37.57227	23.06630	80.688	12.0	23.2	29.7	t.P.
2007-02-11T22:42:54.120	37.24628	23.19972	82.961	187.9	72.4	13.4	t.P.
2007-07-30T07:25:58.570	37.61063	22.60387	57.184	112.5	75.8	71.4	t.P.
2008-01-06T05:14:20.880	37.13075	22.69049	77.024	218.0	45.0	20.0	(*14*)
2008-04-04T04:57:44.360	37.61440	22.44797	50.391	196.7	31.5	-70.6	t.P.
2008-06-18T01:58:45.300	37.70667	22.73510	67.381	91.0	45.0	116.0	(*79*), (*33*)
2008-06-30T13:40:11.400	39.39937	20.89344	46.143	173.6	13.2	-81.2	t.P.
2009-01-11T10:50:05.560	37.61606	22.59360	56.785	278.0	12.4	75.8	t.P., (*33*)
2009-03-16T02:44:32.460	37.97039	23.13436	115.632	42.4	68.9	-21.9	t.P.
2009-05-02T15:44:20.200	37.57874	22.76575	69.764	7.1	51.6	-18.6	t.P.
2009-05-13T22:13:09.360	37.69944	22.35814	46.113	79.9	62.2	-83.2	t.P.
2009-06-05T02:36:04.540	37.97297	21.83933	44.788	206.5	43.8	65.0	t.P.
2010-01-14T04:25:11.330	39.23166	22.18051	79.236	248.3	47.2	56.4	t.P., (*33*)
2010-02-15T15:43:12.630	38.07500	23.19311	123.179	254.4	66.1	23.5	t.P.
2010-03-07T14:12:05.350	38.13164	21.97742	47.912	104.2	81.8	64.7	t.P.
2010-03-12T07:23:28.820	37.78372	22.70007	70.073	352.2	60.7	-43.8	t.P.
2010-03-13T05:28:47.600	37.62664	22.59675	55.113	288.9	16.1	29.2	t.P.
2010-04-19T01:35:41.130	37.71757	23.39209	117.094	224.9	58.4	57.8	t.P.
2010-06-27T13:10:11.630	37.53993	22.87360	68.401	203.1	73.2	-22.7	t.P.
2010-09-06T19:20:43.580	37.61359	22.52836	50.861	242.4	30.5	-78.1	t.P., (*33*)
2010-10-23T23:23:23.160	37.58297	22.61037	56.024	317.3	22.1	34.7	t.P.
2010-12-08T11:37:04.320	38.10997	22.96618	96.757	94.1	44.3	81.4	t.P.
2010-12-29T20:33:30.740	37.90028	22.92929	85.113	207.2	48.0	18.0	t.P.
2011-04-19T12:13:19.640	38.10921	22.01292	48.980	106.7	84.0	87.0	t.P., (*33*)
2011-05-18T14:28:02.050	37.75662	23.41378	129.551	17.7	71.6	-14.0	t.P.
2011-05-21T12:10:43.370	37.61410	22.59712	56.155	356.8	47.2	73.5	t.P.
2011-05-21T23:16:33.100	39.36115	22.26029	91.995	353.8	41.1	48.2	t.P.
2011-06-01T02:10:07.790	38.11387	22.00316	48.685	190.7	33.6	25.5	t.P.
2011-06-07T21:56:14.480	37.70764	21.82440	47.227	135.0	69.7	75.0	t.P.
2011-07-03T09:57:32.930	38.19763	22.12008	57.339	123.7	75.0	89.0	t.P.
2011-07-14T08:41:56.540	37.82325	22.54015	72.215	239.4	52.7	45.3	t.P., (*33*), (*79*)
2011-07-24T18:12:17.440	37.93090	23.12962	111.141	208.8	74.8	17.5	t.P.
2011-08-19T02:34:43.960	38.10889	22.04869	50.838	52.2	44.4	47.9	t.P.

2011-08-21T06:14:21.140	37.68250	23.22221	107.979	17.4	72.8	10.3	t.P.
2011-09-06T04:14:15.840	37.90833	23.06027	97.651	291.8	41.4	80.9	t.P.
2011-09-22T00:41:48.780	38.26258	22.13655	48.757	294.8	87.3	-65.0	t.P.
2012-02-10T10:26:58.580	37.81202	22.29213	52.880	155.2	47.5	-79.1	t.P.
2012-02-20T12:44:31.730	38.12738	22.87755	92.357	30.3	72.0	-17.6	t.P.
2012-06-23T17:52:30.600	38.15479	21.96701	47.882	141.0	19.0	-98.0	(*33*)
2012-11-11T09:25:02.200	38.63799	22.05844	68.927	269.2	38.2	-52.7	t.P.
2012-11-13T11:00:39.280	37.67358	21.87896	49.577	244.3	84.3	-70.9	t.P.
2013-04-12T04:06:59.900	37.62747	22.59445	56.446	247.4	57.2	-15.8	t.P.
2013-04-14T19:41:48.720	38.30219	22.11913	53.962	335.4	10.1	84.3	t.P.
2013-04-28T04:49:56.240	38.26491	22.15148	50.641	123.4	9.1	84.7	t.P. , (*33*)
2013-04-28T16:31:05.730	37.45544	22.63733	54.957	251.5	66.8	-47.9	t.P. , (*33*)
2013-06-26T08:18:04.710	38.10057	22.05250	49.043	347.2	74.5	-32.9	t.P.
2013-07-06T10:10:25.360	39.38246	22.26299	94.221	335.9	49.8	41.3	t.P.
2013-09-21T03:58:40.200	38.02575	23.48732	152.887	147.1	46.1	85.8	t.P.
2013-09-27T02:15:05.000	37.81068	22.08351	58.671	96.9	63.3	70.9	t.P.
2013-11-25T15:10:11.700	37.95745	22.08488	57.784	63.8	80.5	22.1	t.P. , (*33*)
2013-12-21T05:50:40.150	38.26439	22.13109	49.165	326.5	41.8	-48.9	t.P.
2014-05-27T11:21:32.800	37.55861	22.81803	69.657	22.9	68.6	18.5	t.P.
2014-06-25T01:29:14.990	38.10071	22.03666	49.772	166.2	86.0	52.9	t.P.
2014-06-26T05:20:38.700	38.10728	22.04878	49.340	237.0	29.4	-43.0	t.P.
2014-10-28T09:32:58.920	39.10487	22.32926	77.040	56.5	89.6	-23.0	t.P.
2014-12-10T13:13:05.130	38.26243	22.11324	54.593	208.9	47.1	84.5	t.P.
2015-05-04T00:42:01.570	38.26505	21.60220	28.293	262.9	57.6	-57.5	t.P.
2015-06-02T22:44:39.040	39.20571	22.20979	82.416	227.4	62.2	66.1	t.P.
2015-08-09T21:39:22.360	38.13694	21.97710	48.424	344.3	78.2	-22.2	t.P. , (*33*)
2015-08-31T03:04:02.370	38.26150	22.12297	52.811	329.8	11.2	79.6	t.P.
2015-12-12T22:43:50.880	37.59070	22.85322	70.611	40.5	41.7	65.5	t.P.
2016-02-07T00:48:27.740	37.61626	22.60150	55.007	160.0	74.0	90.0	t.P.
2016-04-07T05:56:22.600	38.66357	22.43353	75.630	227.9	56.6	23.4	t.P.
2016-07-17T09:50:21.680	37.62945	22.60436	56.593	132.4	85.2	75.0	t.P.
2016-08-17T09:32:27.770	37.67763	21.89436	50.461	302.0	24.3	80.2	t.P.
2016-11-25T18:07:48.000	37.62473	22.61175	56.422	131.6	65.7	68.0	t.P.
2017-03-03T04:56:22.760	38.12599	21.95401	45.238	294.5	56.0	33.1	t.P.
2017-03-24T04:24:32.560	37.65026	23.81716	156.986	10.0	58.0	-50.0	(*33*)
2017-06-12T18:40:19.080	37.62393	22.59482	65.606	214.7	21.1	47.9	t.P.

Table S3. Locations of mantle wedge seismicity displayed in cross sections in Fig. 4. Listed here are the coordinates of five cross sections from a global selection of cold subduction zones for which precise hypocenter data is available. The width indicates the maximum distance away from the cross section for which earthquakes are plotted in Fig. 4.

**Cross section**	**Region**	**Start of cross section**	**End of cross section**	**Width of cross section**	**Data Source**
A	W Greece: Tripoli	37.20 ºN, 21.77 ºE	38.09 ºN, 23.74 ºE	±25 km	This paper & (*33*)
B	SE Greece: Crete	33.97 ºN, 25.62 ºE	37.55 ºN, 25.62 ºE	±35 km	(*23*, *33*, *59*)
C	NE Japan: Sanriku	38.98 ºN, 142.86 ºE	39.40 ºN, 140.74 ºE	±25 km	(*20*)
D	NE New Zealand: Raukumara	37.52 ºS, 176.99 ºE	38.19 ºS, 178.34 ºE	±17.5 km	(*21*)
E	Lesser Antilles: Martinique	15.35 ºN, 59.65 ºE	14.75 ºN, 61.05 ºE	±23.5 km	(*22*, *61*)

External Data file S1. Deep earthquake hypocenters in Greece.

Link: https://doi.pangaea.de/10.1594/PANGAEA.894348

List of relocated hypocenters of 2172 deep earthquakes of western Greece. The columns in the list contain information on the earthquake's time, latitude, longitude, depth, local magnitude, number of observations (number of P plus S-picks), the horizontal distance along the dip direction in km (x), the horizontal distance along the strike direction in km (y), error in x-direction (km), error in y-direction (km), error in depth (km), relative error in x-direction (km), relative error in y-direction (km), relative error in depth, logical indicator whether the hypocenter stems from double-difference relocation, distance from the slab top in km (positive is above the slab top).

External Data file S2. Deep earthquake focal mechanisms in Greece.

Link: https://doi.pangaea.de/10.1594/PANGAEA.894349

List of preferred focal mechanism solutions for 85 deep earthquakes of western Greece. The list columns contain information on the earthquake's time, latitude, longitude, depth (km), and the focal mechanism's strike, dip, and rake (all in degree), and the source of the solution (where HalpaapEtAl2018 indicates this paper).

External Data file S3. Earthquake arrival time picks.

Link: https://doi.pangaea.de/10.1594/PANGAEA.894350

Dataset containing all measurements required to solve hypocenter locations, magnitude and focal mechanisms. These measurements include the arrival time picks, amplitude measurements, and first arrival polarities for all 2172 deep earthquakes. The file is provided in Nordic format (*80*) and in QuakeML format (*81*).

External Data file S4. Model of the subduction plate interface.

Link: https://doi.pangaea.de/10.1594/PANGAEA.894350

Model of the plate interface between the subducting Ionian plate (i.e. the oceanic part of the African) and the overriding Aegean plate. The model is provided in netCDF format and contains a grid of longitude, latitude, and depth-values that describe the three-dimensional interface. This model is based on the plate interface model published in ref. (*16*). Here we corrected the model slightly such that the clusters of interface earthquakes fall precisely on the interface model. The interplate nature of these earthquakes is supported by their focal mechanisms (thrust solutions) and by their alignment parallel to the plate interface within their respective clusters.

External Data file S5. Thermal structure model of the subduction zone.

Link: https://doi.pangaea.de/10.1594/PANGAEA.894350

Temperature model for the present-day Western Hellenic Subduction Zone, as displayed in Fig. 3A. The model is provided in netCDF format and contains a grid of distance along the dip direction (x, in km), depth (km) and temperature-values (ºC) that describe the two-dimensional temperature field. The creation of this model is described in the "Thermal and phase stability Modeling" section in the methods.
